# Discovery and characterization of sweetpotato’s closest tetraploid relative

**DOI:** 10.1111/nph.17991

**Published:** 2022-02-08

**Authors:** Pablo Muñoz‐Rodríguez, Tom Wells, John R. I. Wood, Tom Carruthers, Noelle L. Anglin, Robert L. Jarret, Robert W. Scotland

**Affiliations:** ^1^ Department of Plant Sciences University of Oxford South Parks Road Oxford OX1 3RB UK; ^2^ Royal Botanic Gardens Kew, Richmond Surrey TW9 3AB UK; ^3^ International Potato Center Avenida La Molina 1895, Distrito de La Molina Lima 15023 Peru; ^4^ United States Department of Agriculture 1109 Experiment Street Griffin GA 30223 USA

**Keywords:** crop wild relatives, Ecuador, genomics, herbarium specimens, *Ipomoea aequatoriensis*, new species, tetraploid

## Abstract

- The origin of sweetpotato, a hexaploid species, is poorly understood, partly because the identity of its tetraploid progenitor remains unknown. In this study, we identify, describe and characterize a new species of *Ipomoea* that is sweetpotato’s closest tetraploid relative known to date and probably a direct descendant of its tetraploid progenitor.
- We integrate morphological, phylogenetic, and genomic analyses of herbarium and germplasm accessions of the hexaploid sweetpotato, its closest known diploid relative *Ipomoea trifida*, and various tetraploid plants closely related to them from across the American continent.
- We identify wild autotetraploid plants from Ecuador that are morphologically distinct from *Ipomoea batatas* and *I. trifida*, but monophyletic and sister to *I. batatas* in phylogenetic analysis of nuclear data.
- We describe this new species as *Ipomoea aequatoriensis* T. Wells & P. Muñoz sp. nov., distinguish it from hybrid tetraploid material collected in Mexico; and show that it likely played a direct role in the origin of sweetpotato’s hexaploid genome. This discovery transforms our understanding of sweetpotato’s origin.

## Abstract 2

See also the Commentary on this article by Särkinen *et al.*, **234**: 1107–1108.

## Introduction

Sweetpotato, *Ipomoea batatas* (L.) Lam., is a hexaploid species thought to have originated via allopolyploidy from a diploid and a tetraploid ancestor (Yang *et al*., 2017). *Ipomoea trifida* (Kunth) G. Don, a Circum‐Caribbean species, was recently confirmed as sweetpotato’s closest diploid relative and most likely the direct descendant of its diploid progenitor (Muñoz‐Rodríguez *et al*., 2018). In contrast, the identity of the sweetpotato’s closest tetraploid relative remains unknown. Identifying this entity is key to untangling the evolutionary history of sweetpotato, understanding its contemporary diversity and assembling its large allohexaploid genome.

**Fig. 1:**
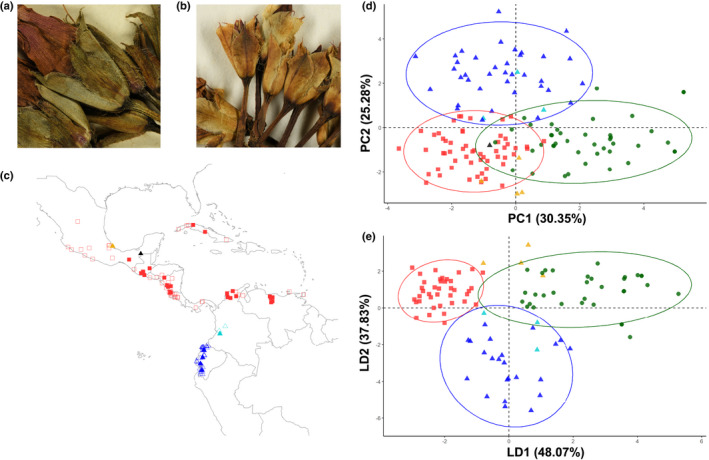
*Ipomoea aequatoriensis* is morphologically distinct from *Ipomoea batatas* and *Ipomoea trifida*. Sepals are (a) oblong/ovate in cultivated *I. batatas* (Balls 5483) and (b) obovate in *I. aequatoriensis* (Jativa and Epling. 1191). (c) Map of the Americas showing the distribution of specimens included in the morphological analysis. Closed symbols indicate specimens also included in the genomic analyses. All hexaploid *I. batatas* specimens in this study are of cultivated origin and are not included in the map. (d) Principal component analysis and (e) linear discriminant analysis of 12 quantitative morphological traits widely used in sweetpotato morphological studies. Ellipses indicate 95% confidence level. In (c–e), *I. batatas* (green dots), *I. aequatoriensis* (blue triangles), *I. trifida* (red squares), hybrids *Ipomoea tabascana* (black triangle) and *I. batatas* var. *apiculata* (orange triangles). The Colombian specimens *affinis* to *I. aequatoriensis* are indicated by light blue triangles.

Whilst preparing a monograph of all American species of *Ipomoea* L. (Wood *et al*., 2020), our attention was drawn to herbarium specimens from Ecuador identified as *I. batatas* but differing in their shorter and blunter sepals (Fig. 1a,b), sepal morphology being an important taxonomic character in *Ipomoea* (Austin, 1978; Wood *et al*., 2020). These specimens were restricted to coastal Ecuador (Fig. 1c) and were of wild provenance, in contrast to most populations of *I. batatas* that are only known from cultivation or as escapes.

As part of our research, in parallel to studying herbarium specimens, we also grew tetraploid *Ipomoea* specimens from seeds available in germplasm collections (Supporting Information Table S1). Tetraploid collections (2*n* = 4*x* = 60) are of particular interest because their ploidy is intermediate between hexaploid sweetpotato (2*n* = 6*x* = 90) and its closest diploid relative, *I. trifida* (2*n* = 2*x* = 30), meaning that they may represent intermediate stages in sweetpotato evolution. The germplasm material studied by us included other tetraploid specimens from the same areas of Ecuador as the distinctive herbarium material we had identified during our studies (Figs S1–S5), as well as material of the Mexican sweetpotato variety *I. batatas* var. *apiculata* J.A. McDonald & D.F. Austin and the Mexican hybrid species *Ipomoea tabascana* J.A. McDonald & D.F. Austin, both of them also tetraploids (Notes S1). *Ipomoea tabascana* is a modern hybrid between *I. batatas* and *I. trifida* known from a single collection (McDonald & Austin, 1990; Srisuwan *et al*., 2006). Modern tetraploid hybrids such as this may confound data interpretation, hence the importance of including in our study examples of known hybrid origin: it is essential to be able to distinguish between truly autotetraploid entities and other tetraploids of modern hybrid origin.

To place the Ecuadorian specimens in a phylogenetic context, we conducted a preliminary phylogenetic analysis using *rpl32‐trnL*, a small, noncoding, rapidly‐evolving chloroplast DNA region. *Ipomoea batatas* contains two different chloroplast lineages (Roullier *et al*., 2013), the ancestral lineage (chloroplast lineage 1) and a second, more recent lineage that is most likely the result of introgression with chloroplast capture from *I. trifida* (lineage 2) (Muñoz‐Rodríguez *et al*., 2018). The preliminary analysis of this small chloroplast DNA region showed that the herbarium specimens and the germplasm material from Ecuador were the same entity, and that they were more closely related to sweetpotato chloroplast lineage 1 than to any other lineage (Methods S1; Fig. S6). A subsequent literature review showed that we were not the first to recognize these tetraploids from Ecuador (Martin & Jones, 1972; Martin *et al*., 1974; Austin *et al*., 1993), but previous studies lacked the taxonomic and phylogenetic framework required to accurately infer their relationship with sweetpotato.

Here, we provide the first comprehensive study of these Ecuadorian tetraploids and show that they represent a distinct species that is sweetpotato’s closest wild relative. We describe this new species as *Ipomoea aequatoriensis* T. Wells & P. Muñoz and show it is most likely the direct descendant of the sweetpotato’s tetraploid progenitor.

## Materials and Methods

### Herbarium collections and germplasm material

We studied American material from germplasm collections (CIP and USDA) and herbaria (AAU, BM, E, FL, FTG, GUAY, HUEFS, K, LPB, OXF, QAC, QAP, QCA, QCNE, RB, ST, US, USZ, XAL, acronyms according to Thiers (2018)). We included specimens of cultivated hexaploid *I. batatas* (L.) Lam. and diploid *I. trifida* (Kunth) G. Don from across their geographical distribution; representatives of the 14 other close wild relatives of sweetpotato (Wood *et al*., 2020) including the Mexican hybrid tetraploid *I. tabascana* J.A. McDonald & D.F. Austin, known from a single collection (Notes S1) (McDonald & Austin, 1990; Austin *et al*., 1991); two specimens of the tetraploid sweetpotato variety *I. batatas* var. *apiculata* J.A. McDonald & D.F. Austin, also from Mexico and apparently restricted to the vicinity of the city of Veracruz (Notes S1); the tetraploid material from Ecuador; a tetraploid accession from Colombia; and multiple herbarium collections of wild plants resembling the tetraploid Ecuadorian material and collected in the same geographical area (Dodson & Gentry, 1978; Austin, 1982; Dodson *et al*., 1985; McDonald & Austin, 1990; Wood *et al*., 2020). See Tables S2 and S3 for passport data of all specimens and indication of analyses they were included in.

### Quantitative morphological analyses

#### Character selection and measurement

We identified and analysed herbarium specimens and germplasm material of *I. trifida* (57 specimens), *I. batatas* (55 specimens), the Ecuadorian tetraploids (44 specimens), *I. tabascana* (one specimen) and *I. batatas* var. *apiculata* (five specimens) (Table S2). We measured 12 morphological characters found to be informative in taxonomic treatments of *Ipomoea* or commonly used to study sweetpotato germplasm collections (Table S2) (Austin, 1978; Huaman, 1991; Wood *et al*., 2020). Measurements were taken using digital callipers or, in the case of digitized herbarium specimens, using the biological‐image analysis software Fiji (Schindelin *et al*., 2012).

#### Clustering analyses

We first ran a principal component analysis (PCA) to investigate phenotypic clustering between *I. batatas*, *I. trifida* and the various tetraploid entities. We used factominer package v.2.4 (Lê *et al*., 2008) in R and divided the tetraploid material into three groups based on geographical distribution and past determinations: (1) Ecuadorian, (2) *I. tabascana*, and (3) *I. batatas* var. *apiculata*. We then used R package mass v.7.3.54 (Venables *et al*., 2002) to assess how well individual specimens could be classified into their assigned groups through a linear discriminant analysis (LDA). We plotted the results of both analyses using the ggplot2 package v.3.3.5 (Wickham, 2016), with ellipses depicting 95% confidence level added using the *stat_ellipse* function.

### Analysis of genomic data

We sequenced 13 new specimens using Illumina whole genome sequencing and incorporated them in our previously‐existing dataset of sweetpotato crop wild relatives (CWRs) (Muñoz‐Rodríguez *et al*., 2018) (Table S2). This material included six Ecuadorian tetraploids (PI 561246, PI 561248, PI 561255, PI 561258, K300/CH71.3, CH81.2), one Colombian tetraploid (K500/CH80.3), diploid *I. trifida* specimens from Colombia (*F. de la Puente 1054*) and Mexico (*F. de la Puente 2961*), three *I. batatas* var. *apiculata* (*D.F. Austin 7480*, PI 518474 and K233) and one *I. tabascana* (PI 518479).

#### DNA processing and sequencing

We extracted DNA using the Plant Tissue Mini protocol for Qiagen DNEasy Plant Mini Kit. We created genomic libraries using the NEBNext Ultra DNA Library Prep Kit for Illumina v.3.0 (New England BioLabs, Ipswich, MA, USA). Sequencing was done at Novogene facilities in Cambridge, UK, using Illumina NovoSeq6000. We obtained 150 bp paired end whole genome data, on average 11 Gb per sample. We filtered the sequence files using default parameters in trimmomatic (Bolger *et al*., 2014) and checked the quality of the reads using FastQC. We used default settings in BBtools’ tadpole (https://sourceforge.net/projects/bbmap/) to correct the reads.

#### Assembly of single copy nuclear regions for phylogenetic analysis

We assembled 386 putative single copy nuclear DNA regions of all samples using a reference‐guided assembly. A detailed description of how these nuclear regions were identified is provided in Methods S2. We mapped the reads to the reference 386 nuclear probes using BBMap (*paired only = t*, *local = t*). We used SAMtools (Danecek *et al*., 2021) to extract all reads mapped to the reference probes and to remove duplicate reads, and Picard Tools (http://broadinstitute.github.io/picard) to realign the reads mapped around indels. We used BCFtools (Danecek *et al*., 2021) for variant calling, indel normalization and variant filtering, and VCFtools’ *vcf‐sort* (Danecek *et al*., 2011) to sort the VCF files.

#### Phylogenetic analysis of nuclear DNA regions

We used phylogenetic analysis of nuclear data to confirm the close relationship between the Ecuadorian tetraploids and sweetpotato. We used consensus sequences and included the tetraploids from Ecuador and Colombia, 10 *I. batatas* specimens, 10 *I. trifida* specimens, one *I. tabascana* specimen, two *I. batatas* var. *apiculata* specimens, one specimen of each of the other 14 species closely related to sweetpotato and one *I. cryptica* J.R.I. Wood & Scotland as outgroup (Muñoz‐Rodríguez *et al*., 2018, 2019). We obtained consensus sequences from VCF variant files using BCFtools *consensus* (Danecek *et al*., 2021) and masked all positions in the consensus sequences with read coverage lower than 5×. The use of consensus sequences in phylogenetic analysis can obscure potential subgenome differentiation in polyploids. However, the lack of a reference genome makes it impossible to assign the alleles in the nuclear regions to specific subgenomes. To minimize the potential effects of divergent subgenomes, we only included likely homozygous variant positions in this analysis. Heterozygous sites were therefore masked and not considered in the main phylogenetic analysis but were included in additional phylogenetic analyses (Methods S3).

We used the biopython script *sequence_cleaner* to remove sequences shorter than 500 bp or with more than 10% ambiguous sites. We excluded three further regions of the analysis (*solyc06g073230.2.1_1*, *solyc08g043170.2.1_1* and *solyc11g012820.1.1_1*) as none of the sequences in those regions passed the filters, as well as one *I. batatas* var. *apiculata* herbarium specimen (*D*.*F. Austin 7480*) with almost 80% missing data.

We aligned each of the regions independently using Mafft v.7.310 (Katoh & Standley, 2013) and removed poorly aligned regions using Gblocks (*half gaps*) (Castresana, 2000; Talavera & Castresana, 2007). We generated summary files of all edited alignments using Amas (Borowiec, 2016) (Table S4). A further 12 alignments that had no variable sites were excluded, so this analysis was done using 371 putative single‐copy nuclear DNA regions. We also used Amas to concatenate the alignments.

We inferred three different phylogenies: (1) partitioned maximum likelihood (ML) analysis of concatenated alignments with automated model selection + merge in Iq‐Tree v.1.6.12 (Nguyen *et al*., 2015; Kalyaanamoorthy *et al*., 2017); (2) Approximate ML analysis of unpartitioned concatenated alignments in multi‐threaded double‐precision FastTree v.2.1.10^54,71^ (GTR + gamma model); and (3) independent gene tree inference using Iq‐Tree v.1.6.12 with automated model selection followed by species tree inference using the coalescent in Astral III (Zhang *et al*., 2018). We used the GNU parallel tool (Tange, 2011) to parallelize and speed up several steps in the pipeline.

#### Principal component analysis

We conducted a PCA of *I. batatas*, *I. trifida*, the Ecuadorian tetraploids, the Colombian tetraploid specimen K500/CH81.3 and the hybrids *I. tabascana* and *I. batatas* var. *apiculata*. We used a subset of 20 *I. batatas* and 20 *I. trifida* samples to try to minimize bias due to uneven population sizes compared to the other entities (Privé *et al*., 2020). We also conducted additional analyses including all *I. batatas* and *I. trifida* samples instead of a subset (Methods S4).

We mapped the nuclear reads to a sweetpotato sample (accession CIP 400435) and called variants using the same procedure as earlier. We filtered out all variants with coverage lower than 5× and ran a linkage disequilibrium pruning step using Plink (‐‐indep‐pairwise 50 10 0.1). We then used Plink (‐‐double‐id ‐‐allow‐extra‐chr ‐‐set‐missing‐var‐ids @:# ‐‐make‐bed ‐‐pca ‐‐geno 0.20 ‐‐snps‐only ‐‐max‐alleles 2) (Chang *et al*., 2015; Purcell, 2021) for the PCA and plotted the results using tidyverse (Wickham *et al*., 2019) and ggplot2 (Wickham, 2016) in Rstudio (RStudio Team, 2021). This analysis used 419 single nucleotide polymorphisms (SNPs) from across the 386 *Ipomoea* nuclear regions, both homozygous and heterozygous.

#### K‐mer analyses

We used GenomeScope2.0 (Ranallo‐Benavidez *et al*., 2020) to assess heterozygosity from k‐mer frequencies of raw, unaligned sequencing reads, in a representative Ecuadorian sample (PI 561248) sequenced at high‐coverage. Relative frequency patterns can then be used to infer whether a tetraploid sample is autopolyploid or allopolyploid. We carried out initial k‐mer counting and histogram construction on the filtered but unaligned sequencing reads using Jellyfish (Marçais & Kingsford, 2011). We ran both Jellyfish and GenomeScope2.0 with a maximum coverage of 100 000 and the default k‐mer value of 21. We also ran the same analysis in three Mexican hybrid tetraploids sequenced at lower coverage (Methods S5).

#### Assembly of whole chloroplast genomes

We used GetOrganelle (*‐F embplant_pt*; SPAdes options: *"‐‐threads 20 ‐‐only‐assembler ‐k 21,33,55,77,93"*) (Jin *et al*., 2018) to *de novo* assemble the chloroplast genomes of the new samples. When GetOrganelle failed to produce a circular genome assembly in the first attempt, we ran a second attempt using *‐‐reduce‐reads‐for‐coverage INF* and *‐‐max‐reads INF* options. GetOrganelle successfully assembled all samples except one *I. trifida* sample (*F. de la Puente 2961*). To assemble the genome of this one sample, we used a reference‐guided assembly using *I. trifida* (*F. de la Puente 1054*) as reference.

#### Phylogenetic network using chloroplast genomes

This analysis includes all *I. batatas*, *I. trifida* and *I. tabascana* specimens from our previous study, together with the 15 newly sequenced samples. We aligned the whole chloroplast genome sequences using Mafft v.7.310 (FFT‐NS‐2) and removed poorly aligned regions using Gblocks (*no gaps*). We used PopArt (http://popart.otago.ac.nz) to infer a Median Joining Network (reticulation tolerance 0.50 (Bandelt *et al*., 1999)) with 602 segregating sites, 182 of them parsimony‐informative.

## Results

### Morphological differentiation

The tetraploid Ecuadorian and Colombian material form a cluster distinct from *I. batatas* and *I. trifida* in PCA and LDA of 12 morphological characters (Fig. 1e,f). The PCA shows three clusters corresponding to *I. batatas*, *I. trifida* and the Ecuadorian/Colombian material, with some overlap at the margins, predominantly between *I. trifida* and *I. batatas* (Fig. 1e). Hybrid specimens from Mexico, i.e. *I. tabascana* (PI 518479) and *I. batatas* var. *apiculata* (PI 518474), fall close to or within the clusters of *I. trifida* and *I. batatas*. The three distinct clusters were more pronounced in the LDA trained on 80% of the data (Fig. 1e), which yielded a 90% success rate in accurately identifying the test data and recovered the Mexican hybrids within *I. trifida*.

### Genomic differentiation

**Fig. 2:**
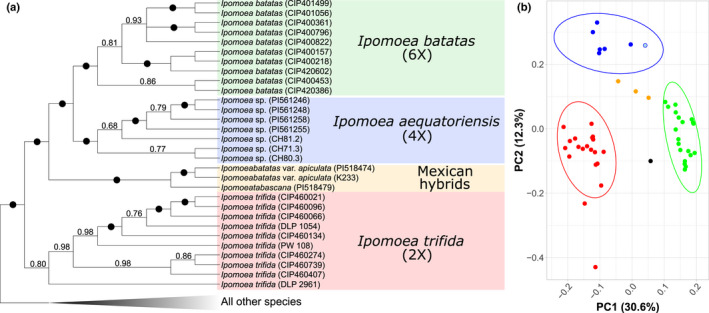
Molecular analyses identify *Ipomoea aequatoriensis* as a distinct entity, phylogenetically distinct and isolated in the genetic space. (a) Approximate maximum likelihood analysis of 371 single‐copy nuclear DNA regions. Numbers on the branches indicate Shimodaira–Hasegawa‐like support values; black dots indicate branches with 100% support. (b) Principal component analysis of *Ipomoea batatas* (green), *Ipomoea trifida* (red), *I*. aequatoriensis (blue) and the hybrids *Ipomoea tabascana* and *I. batatas* var. *apiculata* (black and orange respectively). Principal component analysis inferred using 419 single nucleotide polymorphisms (SNPs) from across the 386 nuclear probes. Ellipses indicate multivariate *t*‐distribution. The Colombian specimen K500/CH81.3 discussed throughout the text is indicated in light blue.

The phylogenetic analysis of nuclear regions recovers the six tetraploid specimens from Ecuador and one from Colombia in a clade sister to hexaploid *I. batatas* (Fig. 2a). This relationship is recovered in all methods of phylogenetic inference with strong support (Figs S7, S8). In addition, the tetraploid Ecuadorian and Colombian specimens also form a distinct group from *I. batatas* and *I. trifida* in the different PCA using nuclear SNPs (Figs 2b, S9). The analysis using a subset of *I. batatas* and *I. trifida* samples, shown in Fig. 2(b), aimed at preventing bias due to uneven population sizes (Privé *et al*., 2020). In this analysis, the Ecuadorian and Colombian tetraploids partially overlap with *I. trifida* in principal component one (PC1) but clearly separate from all entities, including *I. trifida*, in principal component two (PC2). The single specimen of the Mexican hybrid *I. tabascana* and the three *I. batatas* var. *apiculata* specimens are intermediate between *I. trifida* and *I. batatas* in PC1 but cluster with both species in PC2 (Fig. 2b).

The analysis of nucleotide heterozygosity patterns suggests that the Ecuadorian tetraploids have a genomic structure consistent with an autopolyploid origin, with proportions of *aaab* consistently higher than *aabb* (Table S5; Notes S1). This pattern is indicative of two identical or highly similar subgenomes originating from a whole genome duplication (Ranallo‐Benavidez *et al*., 2020). The same analysis for the hybrid *I. tabascana* and two specimens of *I. batatas* var. *apiculata*, albeit using lower coverage data (Methods S2), shows instead a higher proportion of *aabb* than *aaab* (Table S5; Notes S1), which suggests that two distinct subgenomes have been derived from a recent hybridization event.

### Analysis of whole chloroplast genomes

The Median Joining phylogenetic network inferred using 602 segregating sites from the alignment of whole chloroplast genomes shows the Ecuadorian plants are associated with the ancestral sweetpotato lineage 1, whereas the single Colombian specimen we sequenced (K500/80.3) is associated with the sweetpotato lineage 2. The hybrid *I. tabascana* and *I. batatas* var. *apiculata* are also associated with sweetpotato lineage 2.

## Discussion

### *Ipomoea aequatoriensis* is a distinct species

We have identified a group of plants from Ecuador that are distinct from cultivated sweetpotato and from all sweetpotato CWRs known to date. These tetraploid plants are of wild provenance, morphologically and geographically coherent, most likely autotetraploid, isolated in the genetic space, and form a monophyletic group most closely related to sweetpotato in phylogenetic analysis of nuclear data. Their distinctiveness justifies recognition as a new species *I. aequatoriensis* T. Wells & P. Muñoz. A formal diagnosis is presented here. Specimen citation, full description and ecological notes are provided in the Notes S2. Specimens from Colombia, although possibly also part of this species, require further study and are not formally included in *I. aequatoriensis* (see Notes S2).

#### *Ipomoea aequatoriensis* T. Wells & P. Muñoz, sp. nov. (Illustration in Fig. S10)

TYPE: ECUADOR. Esmeraldas Province, Quinindé. *Austin, D.F*. 7803 (holotype FTG, Isotype CIP).

#### Diagnosis

**Fig. 3:**
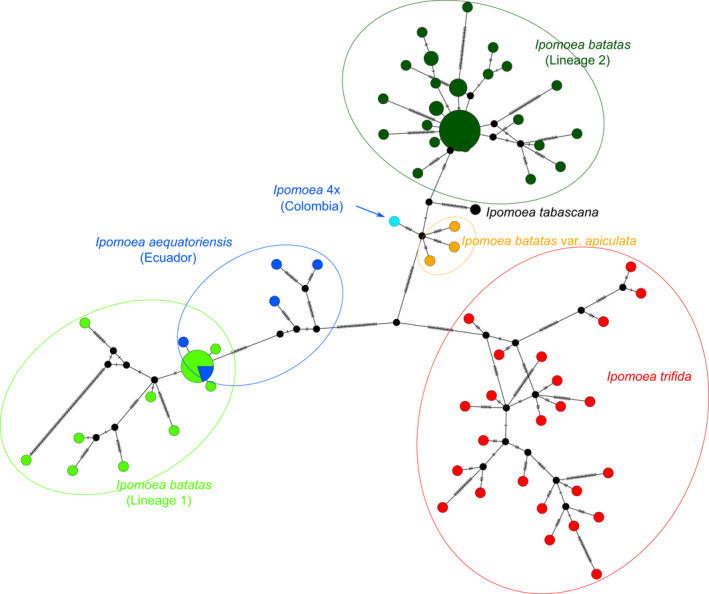
The analysis of chloroplast genomes shows *Ipomoea* aequatoriensis is associated with the sweetpotato ancestral lineage. Median Joining phylogenetic network inferred using 602 segregating sites (182 parsimony‐informative) and showing the relationships between *Ipomoea batatas*, *Ipomoea trifida*, *I. aequatoriensis* and the hybrid entities, *Ipomoea tabascana* and *I. batatas* var. *apiculata*. The one Colombian specimen sequenced (K500/CH80.3), indicated with an arrow, seems to carry a chloroplast related to sweetpotato lineage 2 chloroplast; we excluded it from our diagnosis of *I. aequatoriensis* pending further investigation. The size of the circles indicates the number of samples, with samples grouping in larger circles being identical for the sites studied.

This species is most closely related to *I. batatas* (L.) Lam. (Figs 2a, 3) which it resembles in corolla size, dense sub‐umbellate inflorescence and pubescent ovary, but differs in possessing sepals that are consistently shorter (outer: < 7 vs > 7 mm; inner: < 10 mm vs > 12 mm) and stems that are thinner (1–3 mm vs 2–6 mm diameter) with longer internodes (6–16 cm vs 2–10 cm), consistent with a twining (rather than trailing) habit. It also closely resembles *I. trifida* (Kunth) G. Don, particularly in the twining habit and chartaceous sepals, but differs in having obtuse sepals (80°–160° vs 20°–70°) and laxer, more obviously umbellate inflorescences with a greater number of flowers (5–24 vs 2–12) and mostly entire, larger leaves (4–14 cm vs 2–10 cm long).

### Identifying the tetraploid progenitor of sweetpotato

**Fig. 4:**
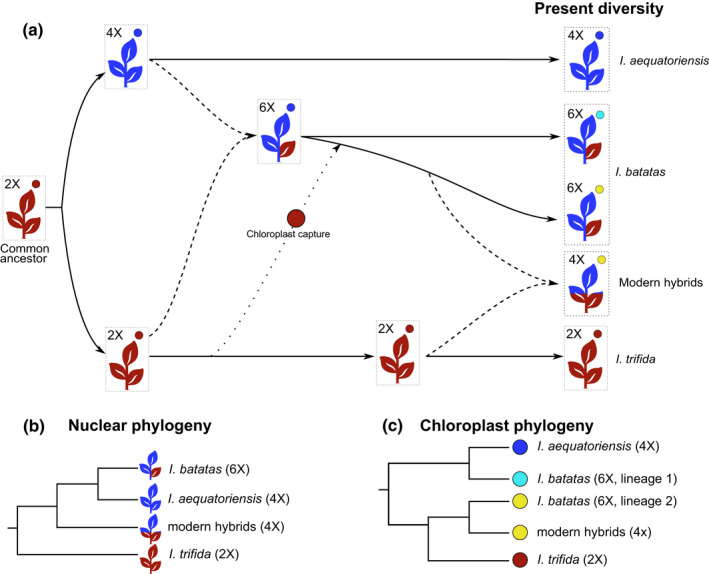
One of several possible scenarios of sweetpotato evolution and origin of current diversity. Tetraploid plants closely related to sweetpotato have two different origins: plants from Ecuador represent direct descendants from the autotetraploid progenitor of hexaploid *Ipomoea batatas*, whereas plants from Mexico and Central America are the result of a more recent hybridization between hexaploid *I. batatas* and diploid *Ipomoea trifida*. (a) One possible scenario, congruent with the data currently available, is presented here. An autotetraploid would have arisen from a whole genome duplication of a diploid common ancestor with *I. trifida*. This autotetraploid would have hybridized with the diploid ancestor to produce an allohexaploid. Subsequent introgression between the diploid ancestor lineage and the allohexaploid would result in chloroplast capture from *I. trifida*, explaining the two distinct *I. batatas* lineages in the chloroplast phylogenies. This separate lineage would keep a hexaploid nuclear genome but a chloroplast most similar to the diploid progenitor, and therefore to modern *I. trifida*, than to the *ancestral* sweetpotato lineage. Red and blue colours indicate the proportion of diploid (AA, red) and tetraploid (BBBB, blue) ancestral genomes in the different entities. Small, coloured circles represent the chloroplast. Dashed lines indicate hybridization and dotted line indicates introgression with chloroplast capture. (b) Summary nuclear phylogeny depicting the relationship between modern taxa, with *Ipomoea aequatoriensis* most closely related to *I. batatas*. (c) Summary chloroplast phylogeny depicting the relationship between modern taxa, with *I. aequatoriensis* most closely related to *I. batatas* lineage 1, the ancestral sweetpotato lineage.

A major barrier to understanding the origin and evolution of sweetpotato remains the difficulty of assembling its large allohexaploid genome (Isobe *et al*., 2019), which comprises three subgenomes: two identical (BBBB) and one slightly different (AA) in an AABBBB structure (Ting & Kehr, 1953; Ting *et al*., 1957; Jones, 1965; Magoon *et al*., 1970; Nishiyama *et al*., 1975; Shiotani & Kawase, 1987; Srisuwan *et al*., 2006; Gao *et al*., 2011; Yang *et al*., 2017). These subgenomes are most likely derived from a hybridization event between a diploid progenitor that contributed the AA subgenome and a tetraploid progenitor that contributed the BBBB subgenomes (Fig. 4). The AA subgenome is most likely derived from a diploid ancestor shared with *I. trifida* (Yang *et al*., 2017; Muñoz‐Rodríguez *et al*., 2018), but the tetraploid progenitor that contributed the BBBB subgenomes remains unidentified (Yang *et al*., 2017).

The new autotetraploid species *I. aequatoriensis* is the closest wild relative of sweetpotato identified to date, and our results strongly suggest it is the direct descendant of sweetpotato’s tetraploid progenitor. A possible scenario for this is presented in Fig. 4, and there are four lines of evidence for this conclusion. First, the wild provenance of the samples we studied, which were not cultivated, feral or derived from breeding programmes (Notes S3). Second, *I*. *aequatoriensis* is consistently recovered as monophyletic and sister to *I. batatas* in nuclear phylogenies, regardless of the method of phylogenetic inference, both in our study (Figs 2a, S7, S8) and in a recent pre‐print (Yan *et al*., 2021). Third, its genetic structure is indicative of an autopolyploid origin (Table S5; Notes S1), a requirement for the tetraploid progenitor of the sweetpotato because of the AABBBB structure of the sweetpotato genome. Fourth, *I. aequatoriensis* is most closely related to sweetpotato lineage 1 – the ancestral sweetpotato lineage – in the analyses of chloroplast genomes in our study (Fig. 3) and that by Roullier *et al*. (2013).

### Poor taxonomy and modern hybrids complicate sweetpotato studies

Previous attempts to identify sweetpotato’s tetraploid progenitor have been hampered by taxonomic confusion, the lack of a well‐resolved phylogenetic framework for sweetpotato and its closest relatives or the inclusion of probably feral specimens (Jones, 1967; Nishiyama, 1971; Martin & Jones, 1972; Austin, 1988; Roullier *et al*., 2013) (Table S1).

In addition, the existence of modern hybrids between *I. batatas* and its closest diploid relative, *I. trifida*, further complicates data interpretation. This is because hybridization between *I. batatas* (3*n* gametes) and *I. trifida* (1*n* gametes) will most likely produce a tetraploid (Orjeda *et al*., 1991), as in the case of *I. tabascana* (Austin, 1977; Jarret *et al*., 1992; Bohac *et al*., 1993; Srisuwan *et al*., 2006). Because of their parentage, such tetraploids are closely related to hexaploid *I. batatas* in nuclear phylogenies (Figs 2a, S7). Therefore, studies that rely purely on phylogenetic analysis of nuclear DNA sequence data are likely to confuse these putative hybrid tetraploids with the autotetraploid progenitor of hexaploid *I. batatas* (Yan *et al*., 2021) (Notes S3). However, the incorporation of other lines of evidence confirms the hybrid origin of these tetraploid entities and shows they cannot be the tetraploid progenitor of sweetpotato. First, *I. batatas* var. *apiculata* is recovered with the known hybrid *I. tabascana* in all phylogenies (Figs 2a, S7, S8) and both entities are in an intermediate position between *I. trifida* and *I. batatas* in the PCAs using nuclear genomic variants (Fig. 2b), implying a highly similar genetic structure. Second, k‐mer analysis of these samples suggests that they possess two distinct subgenomes (Table S5; Notes S1). The k‐mer analyses require confirmation using higher‐coverage sequence data (Methods S2), but our results are consistent with their apparent hybrid origin (Srisuwan *et al*., 2006; Muñoz‐Rodríguez *et al*., 2018). Third, the hybrid entities are most closely related in the chloroplast analysis to the derived sweetpotato chloroplast lineage 2 (Fig. 3), which is the result of introgression with *I. trifida* and therefore postdates the origin of *I. batatas* (Muñoz‐Rodríguez *et al*., 2018). Finally, the PCA and LDA using morphology (Fig. 1d,e) consistently show these specimens cluster with either *I. trifida* or *I. batatas*, instead of forming a distinct group as is the case of *I. aequatoriensis*.

In summary, a broader consideration of collection history, nuclear and chloroplast sequence data, and genomic structure, enables the identification of modern tetraploid hybrids, such as *I. tabascana* and *I. batatas* var. *apiculata*, and rules them out as sweetpotato’s tetraploid progenitor (Fig. 4). Our results also suggest *I. batatas* var. *apiculata* should be treated as a distinct entity of hybrid origin akin to *I. tabascana* rather than a subspecies of *I. batatas* (Notes S1). Although we have not been able to study all the material listed in earlier studies of tetraploid plants (Table S1; Notes S4), future studies that consider the different criteria presented here should allow the classification of those specimens as either ancient autotetraploids or modern hybrids, and further clarify their relationship to sweetpotato.

### *Ipomoea aequatoriensis*, a key finding for sweetpotato studies

The identification of the closest living relative of the tetraploid progenitor of sweetpotato is key to untangling its genomic history and contemporary diversity. *Ipomoea aequatoriensis* has all the hallmarks of being that species, and therefore represents an extraordinary discovery and a key finding for subsequent sweetpotato studies.

## Author contributions

RWS, JRIW, PM‐R, TW and TC conceived the project; PM‐R and TW conducted the analyses; NLA and RLJ contributed material and information about its provenance; PM‐R, TW, RWS, JRIW and TC wrote the manuscript. PM‐R and TW contributed equally to this work.

## Supporting information


**Fig. S1** *Ipomoea aequatoriensis* specimen PI 355830/K300/CH71.3.
**Fig. S2** *Ipomoea aequatoriensis* specimen K500/CH80.3.
**Fig. S3** *Ipomoea aequatoriensis* specimen PI 561248/CIP 403553.
**Fig. S4** *Ipomoea aequatoriensis* specimen PI 561258.
**Fig. S5** *Ipomoea tabascana* specimen *PI 518479/CIP 460824* and *Ipomoea batatas* var. *apiculata* specimen PI 518474/CIP 403953.
**Fig. S6** *trnL‐rpl32* chloroplast DNA barcode phylogeny.
**Fig. S7** Nuclear phylogenies of *Ipomoea* Clade A3 indicating the position of the Ecuadorian tetraploids and the modern hybrids.
**Fig. S8** Nuclear phylogenies of *Ipomoea* Clade A3 indicating the position of the Ecuadorian tetraploids and the modern hybrids. Phylogenies inferred including IUPAC characters for heterozygous sites.
**Fig. S9** Additional principal component analyses.
**Fig. S10** Scientific illustration of *Ipomoea aequatoriensis* T. Wells & P. Muñoz.
**Methods S1** Preliminary analysis of the trnL‐rpl32 chloroplast DNA region.
**Methods S2** K‐mer analysis of putative hybrid tetraploids.
**Methods S3** Additional phylogenetic analysis of nuclear probes.
**Methods S4** Additional principal component analyses.
**Methods S5** K‐mer analysis of putative hybrid tetraploids.
**Notes S1** Modern hybrids closely related to *Ipomoea batatas*.
**Notes S2** K‐mer analyses diagrams.
**Notes S3** Description and additional information for *Ipomoea aequatoriensis*.
**Notes S4** Hybrid specimens in other studies.

**Table S1** Tetraploid accessions in previous studies, indicating past and present identifications.

**Table S2** Passport data of all samples included in morphological analyses.

**Table S3** Passport data of all samples included in phylogenetic analyses.

**Table S4** Statistics of the putative single copy nuclear regions used in phylogenetic analysis.
**Table S5** Patterns of nucleotide heterozygosity in k‐mer spectra of sequencing reads (*k* = 21).
Please note: Wiley Blackwell are not responsible for the content or functionality of any Supporting Information supplied by the authors. Any queries (other than missing material) should be directed to the *New Phytologist* Central Office.

## Data Availability

Raw reads from the 2018 study and newly generated data are available in the Sequence Repository Archive, BioProjects PRJNA453382 and PRJNA796763 respectively. Original and edited files with morphological and molecular analyses and scripts are available via the Oxford Research Archive (https://ora.ox.ac.uk/objects/uuid:055e2f01‐bbb1‐4a69‐a3ae‐dac009db31d1). Any other information required to re‐analyse the data is available from the lead contact upon request.
