# Supplementary material for: Discovery and characterization of sweetpotato’s closest tetraploid relative
Source: New Phytol. 2022 Feb 8;234(4):1185–94. doi: 10.1111/nph.17991 (PMC9306577; doi:10.1111/nph.17991)
Supplement: Supplementary file 1 — Fig. S1 Ipomoea aequatoriensis specimen PI 355830/K300/CH71.3. Fig. S2 Ipomoea aequatoriensis specimen K500/CH80.3. Fig. S3 Ipomoea aequatoriensis specimen PI 561248/CIP 403553. Fig. S4 Ipomoea aequatoriensis specimen PI 561258. Fig. S5 Ipomoea tabascana specimen PI 518479/CIP 460824 and Ipomoea batatas var. apiculata specimen PI 518474/CIP 403953. Fig. S6 trnL‐rpl32 chloroplast DNA barcode phylogeny. Fig. S7 Nuclear phylogenies of Ipomoea Clade A3 indicating the position of the Ecuadorian tetraploids and the modern hybrids. Fig. S8 Nuclear phylogenies of Ipomoea Clade A3 indicating the position of the Ecuadorian tetraploids and the modern hybrids. Phylogenies inferred including IUPAC characters for heterozygous sites. Fig. S9 Additional principal component analyses. Fig. S10 Scientific illustration of Ipomoea aequatoriensis T. Wells & P. Muñoz. Methods S1 Preliminary analysis of the trnL‐rpl32 chloroplast DNA region. Methods S2 K‐mer analysis of putative hybrid tetraploids. Methods S3 Additional phylogenetic analysis of nuclear probes. Methods S4 Additional principal component analyses. Methods S5 K‐mer analysis of putative hybrid tetraploids. Notes S1 Modern hybrids closely related to Ipomoea batatas. Notes S2 K‐mer analyses diagrams. Notes S3 Description and additional information for Ipomoea aequatoriensis. Notes S4 Hybrid specimens in other studies. [file NPH-234-1185-s001.pdf]

***New Phytologist* Supporting Information**

Article title: Discovery and characterisation of sweetpotato's closest tetraploid relative
Authors: Pablo Muñoz-Rodríguez, Tom Wells, John R.I. Wood, Tom Carruthers, Noelle L. Anglin, Robert L. Jarret & Robert W. Scotland
Article acceptance date: 16 January 2022

The following Supporting Information is available for this article:

Fig. S1 *Ipomoea aequatoriensis* specimen PI 355830/K300/CH71.3.

Fig. S2 *Ipomoea aequatoriensis* specimen K500/CH80.3.

Fig. S3 *Ipomoea aequatoriensis* specimen PI 561248/CIP 403553.

Fig. S4 *Ipomoea aequatoriensis* specimen PI 561258.

Fig. S5 *Ipomoea tabascana* specimen *PI 518479/CIP 460824* and *I. batatas* var. *apiculata* specimen PI 518474/CIP 403953.

Fig. S6 *trnL-rpl32* chloroplast DNA barcode phylogeny

Fig. S7 Nuclear phylogenies of *Ipomoea* Clade A3 indicating the position of the Ecuadorian tetraploids and the modern hybrids

Fig. S8 Nuclear phylogenies of *Ipomoea* Clade A3 indicating the position of the Ecuadorian tetraploids and the modern hybrids. Phylogenies inferred including IUPAC characters for heterozygous sites

Fig. S9 Additional Principal Component Analyses

Fig. S10 Scientific illustration of *Ipomoea aequatoriensis* T.Wells & P.Muñoz

Table S1 Tetraploid accessions in previous studies, indicating past and present identifications (Excel file)

Table S2 Passport data of all samples included in morphological analyses (Excel file)

Table S3 Passport data of all samples included in phylogenetic analyses (Excel file)

Table S4 Statistics of the putative single copy nuclear regions used in phylogenetic analysis (Excel file)

Table S5 Patterns of nucleotide heterozygosity in k-mer spectra of sequencing reads (k=21).

Methods S1 Preliminary analysis of the trnL-rpl32 chloroplast DNA region

Methods S2 K-mer analysis of putative hybrid tetraploids

Methods S3 Additional phylogenetic analysis of nuclear probes

Methods S4 Additional Principal Component analyses.

Methods S5 K-mer analysis of putative hybrid tetraploids.

Notes S1 Modern hybrids closely related to *Ipomoea batatas*

Notes S2 K-mer analyses diagrams.

Notes S3 Description and additional information for *Ipomoea aequatoriensis.*

Notes S4 Hybrid specimens in other studies.

Supplementary References

Figure S1. *Ipomoea aequatoriensis* specimen PI 355830/K300/CH71.3. Collected by *C. M. Rick* s.n. (Ecuador, 1970). Seeds received from USDA and grown at University of Oxford Department of Plant Sciences. Photographs by Tom Wells. A: Habit; B: Roots; C: Inflorescence.

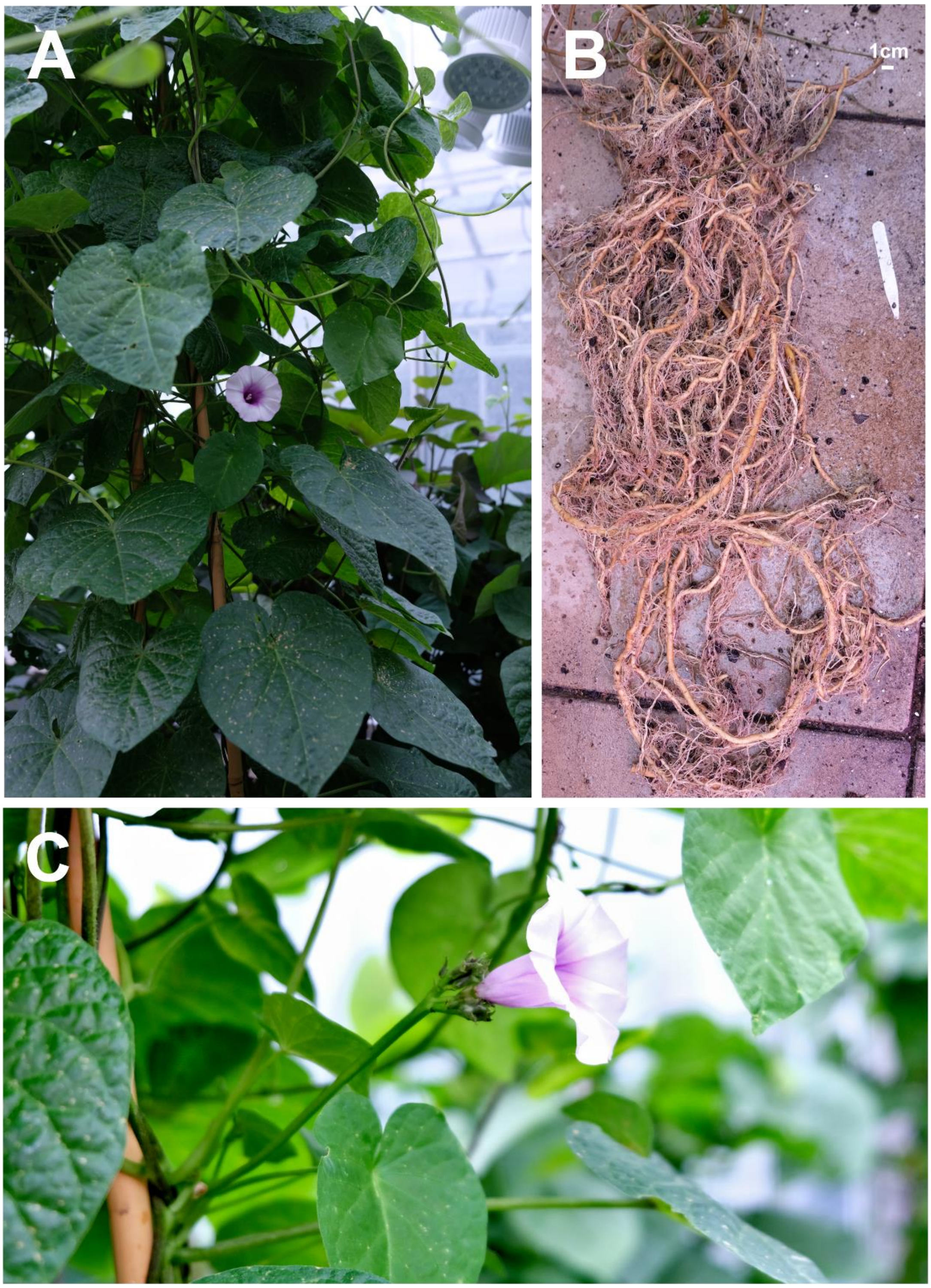

Figure S2. *Ipomoea aequatoriensis* specimen K500/CH80.3: Collected by *M. Kobayashi* s.n. (Colombia, 1976). Seeds received from USDA and grown at University of Oxford Department of Plant Sciences. Photographs by Tom Wells. A: Inflorescence; B: Roots; C: Habit and trilobed leaves.

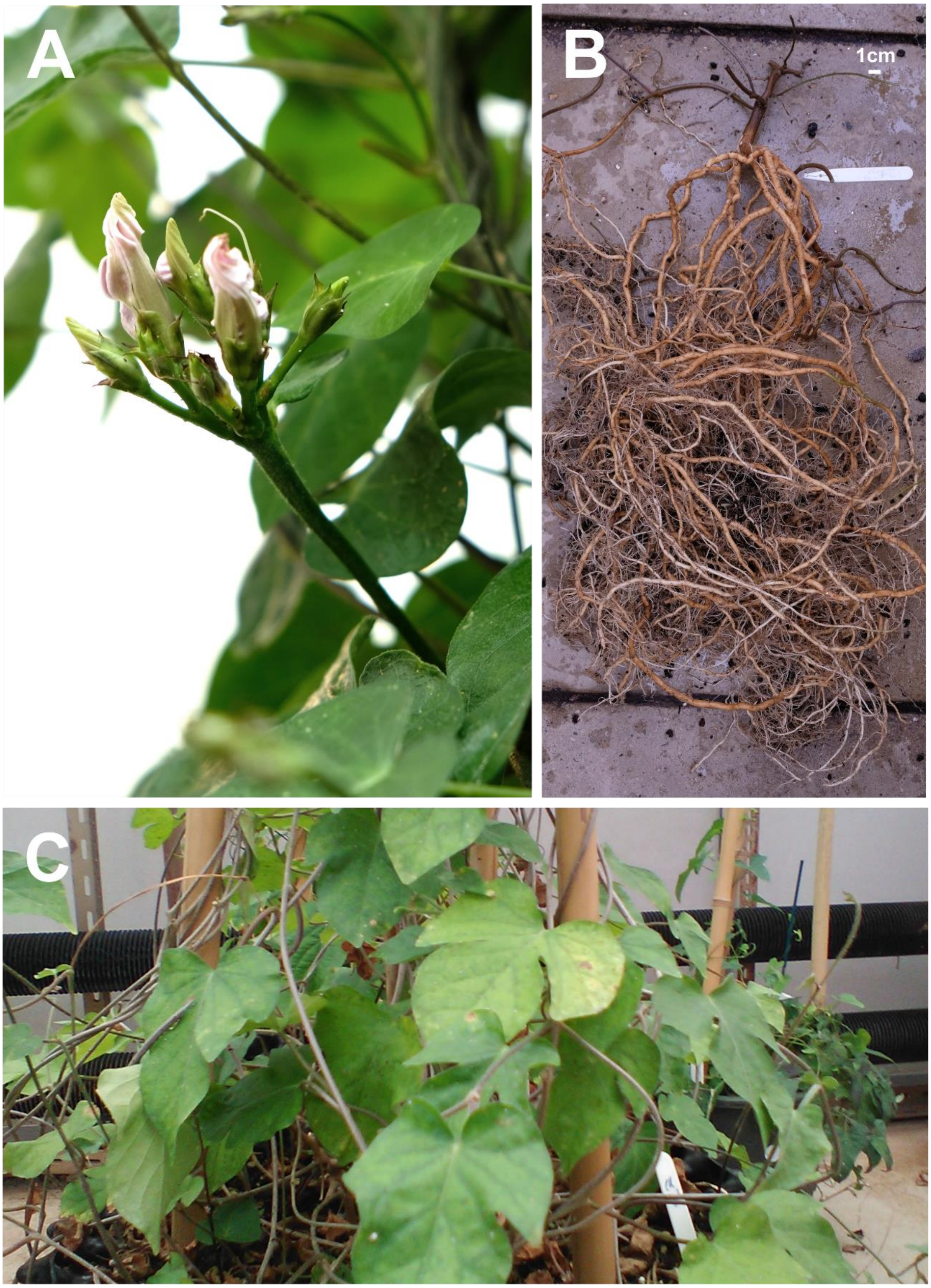

Figure S3. *Ipomoea aequatoriensis* specimen PI 561248/CIP 403553. Collected by *D. F. Austin* 7803 & *F. De La Puente* 5284 (Ecuador, 1992). Seeds received from USDA and grown at University of Oxford Department of Plant Sciences. Photographs by Tom Wells. A: Habit; B: Roots; C: Lobed and unlobed leaves

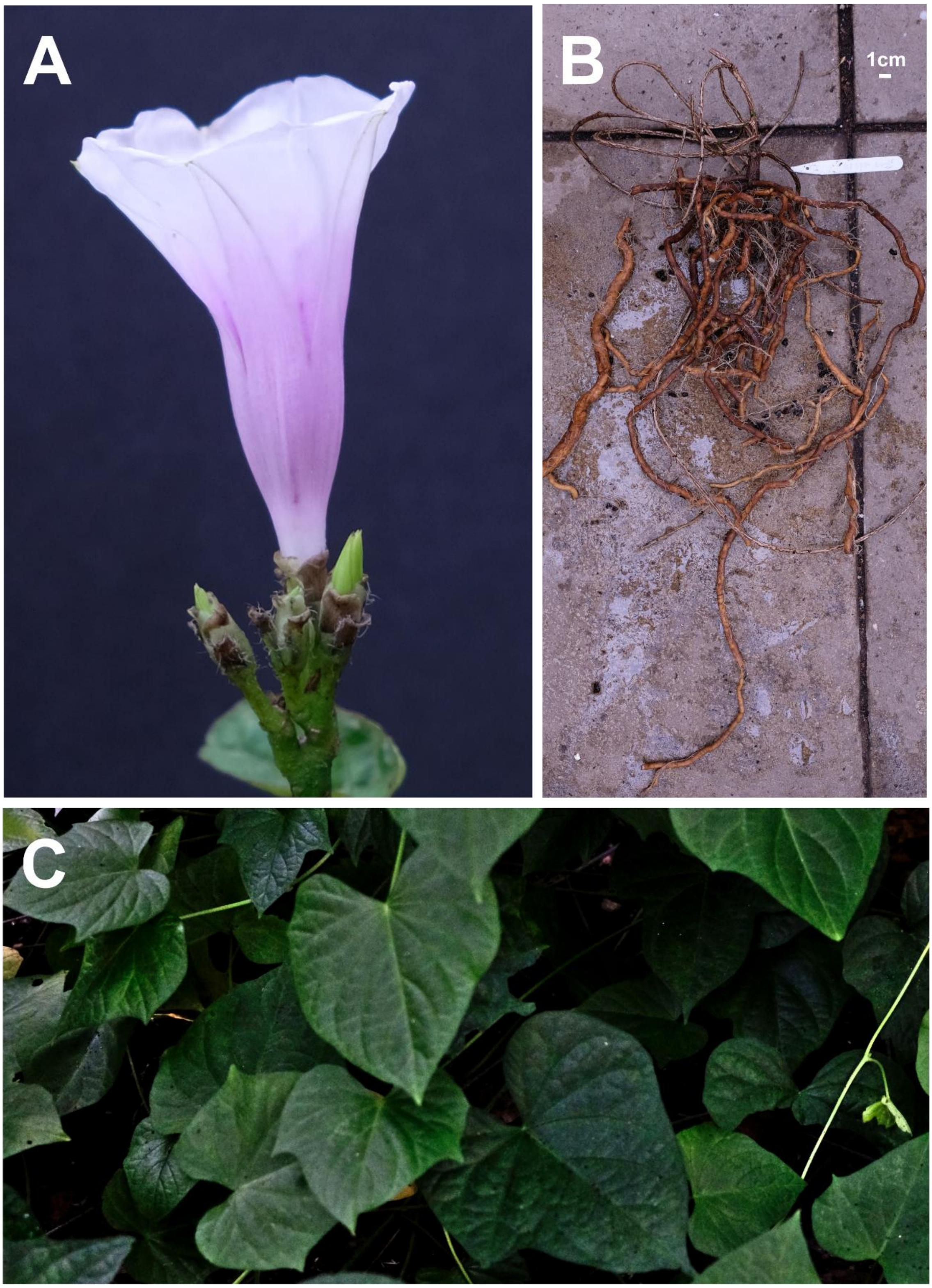

.

Figure S4. *Ipomoea aequatoriensis* specimen PI 561258. Collected by *D. F. Austin* 7817 & *F. De La Puente* 5298 (Ecuador, 1992). Seeds received from USDA and grown at University of Oxford Department of Plant Sciences. Photographs by Tom Wells. **A:** Inflorescence and leaves; **B:** roots; **C:** corolla.

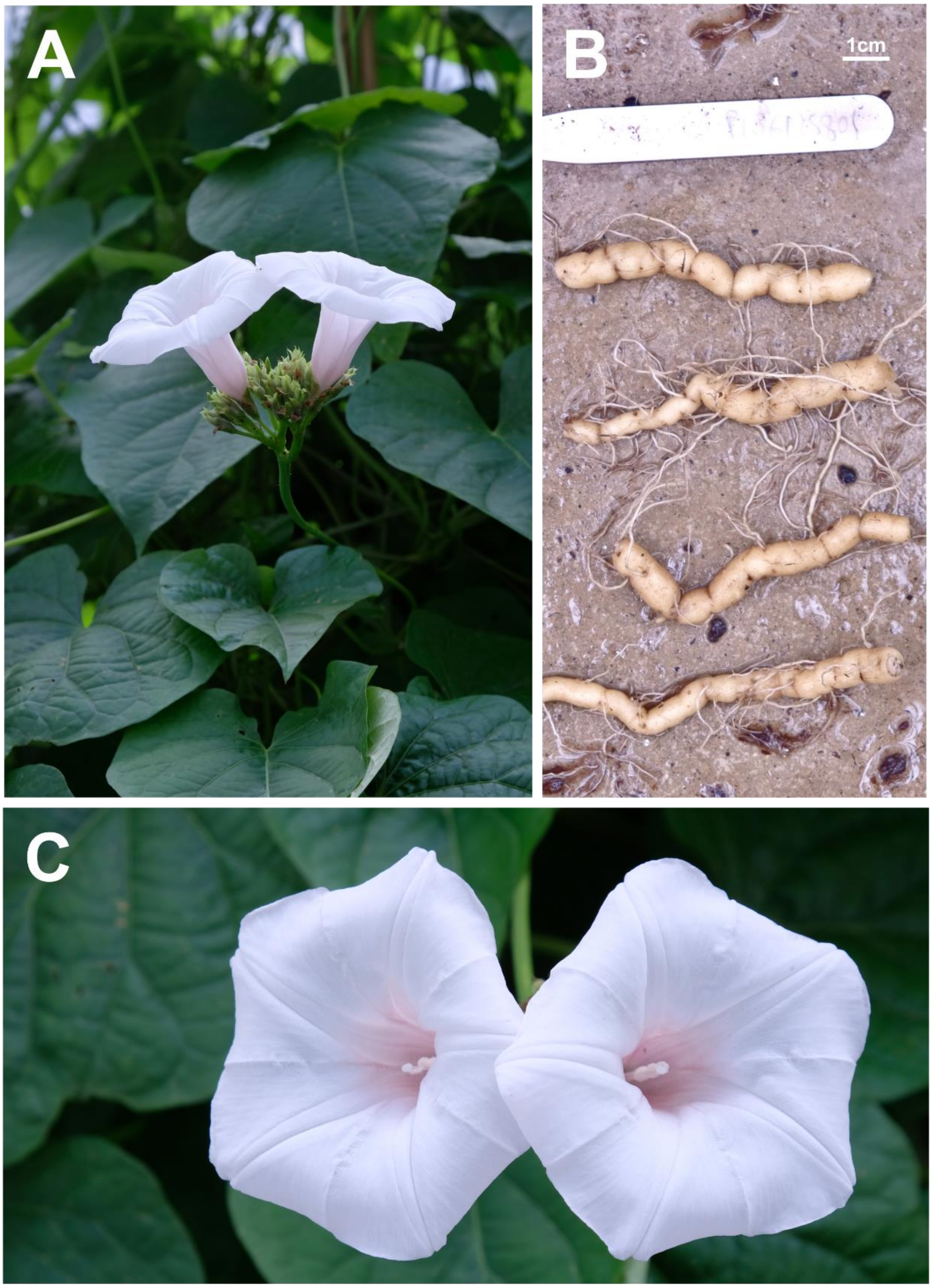

Figure S5. *Ipomoea tabascana* specimen PI 518479/CIP 460824 (A and C) and *I. batatas* var. *apiculata* specimen PI 518474-CIP 403953 (B). *Ipomoea tabascana specimen* collected by *D.F. Austin* 7505 & *F. de la Puente* 2946 (Mexico, 1990). *Ipomoea batatas* var. *apiculata* specimen collected by *F. De La Puente* 2908 (Mexico, 1987). Seeds received from USDA and grown at University of Oxford Department of Plant Sciences. Photographs by Tom Wells and John Baker.

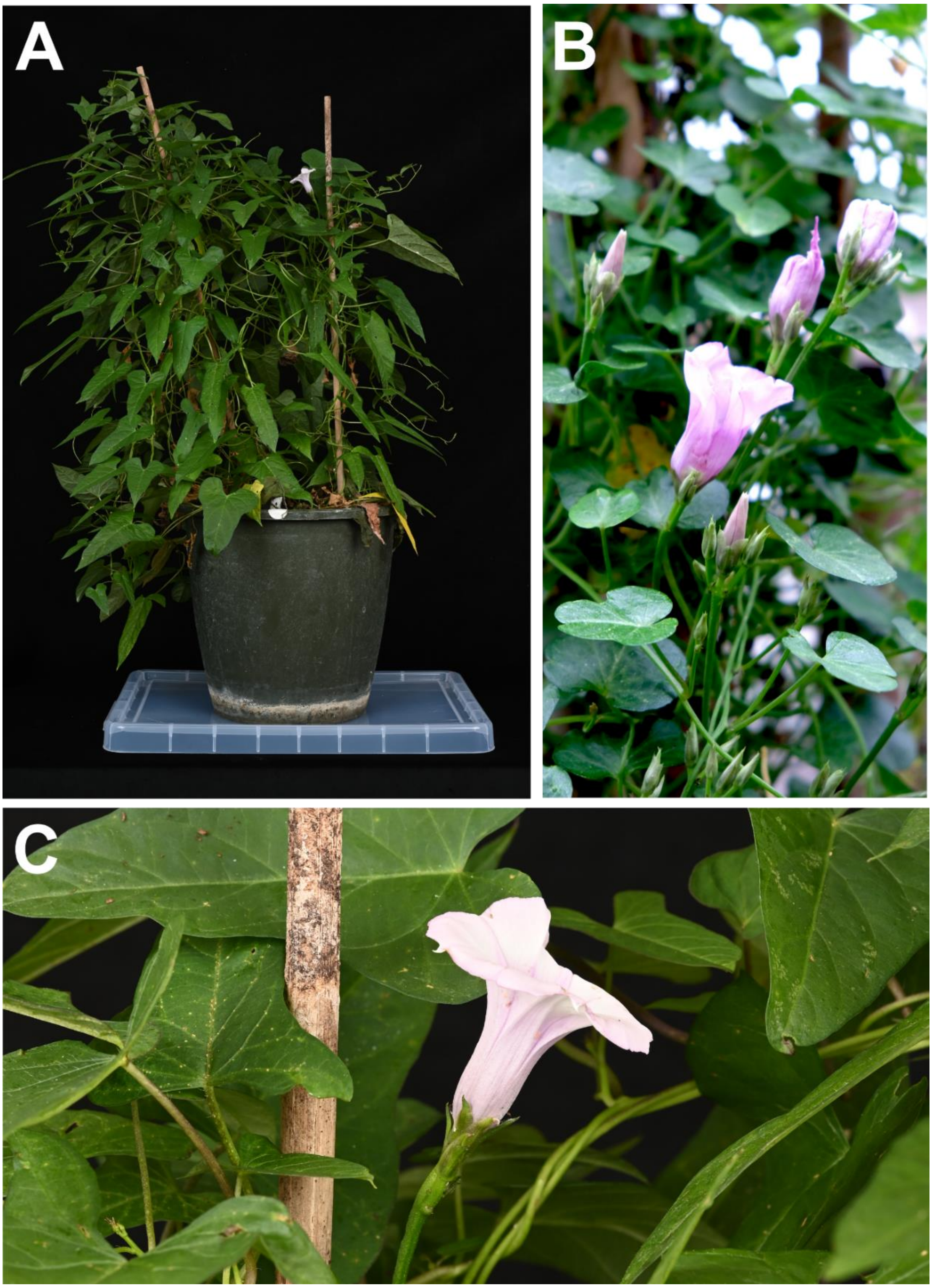

Figure S6. *trnL-rpl32* chloroplast DNA barcode phylogeny of the genus *Ipomoea* inferred using Approximate Maximum Likelihood in FastTree 2. Thick black line indicates the *Ipomoea* clade A3 (also referred to as Batatas clade). Green lines indicate *I. batatas* chloroplast lineage 1 and *I. aequatoriensis* (black crosses for *I. aequatoriensis*)*;* and red lines indicate *I. batatas* lineage 2 with the Mexican hybrids.

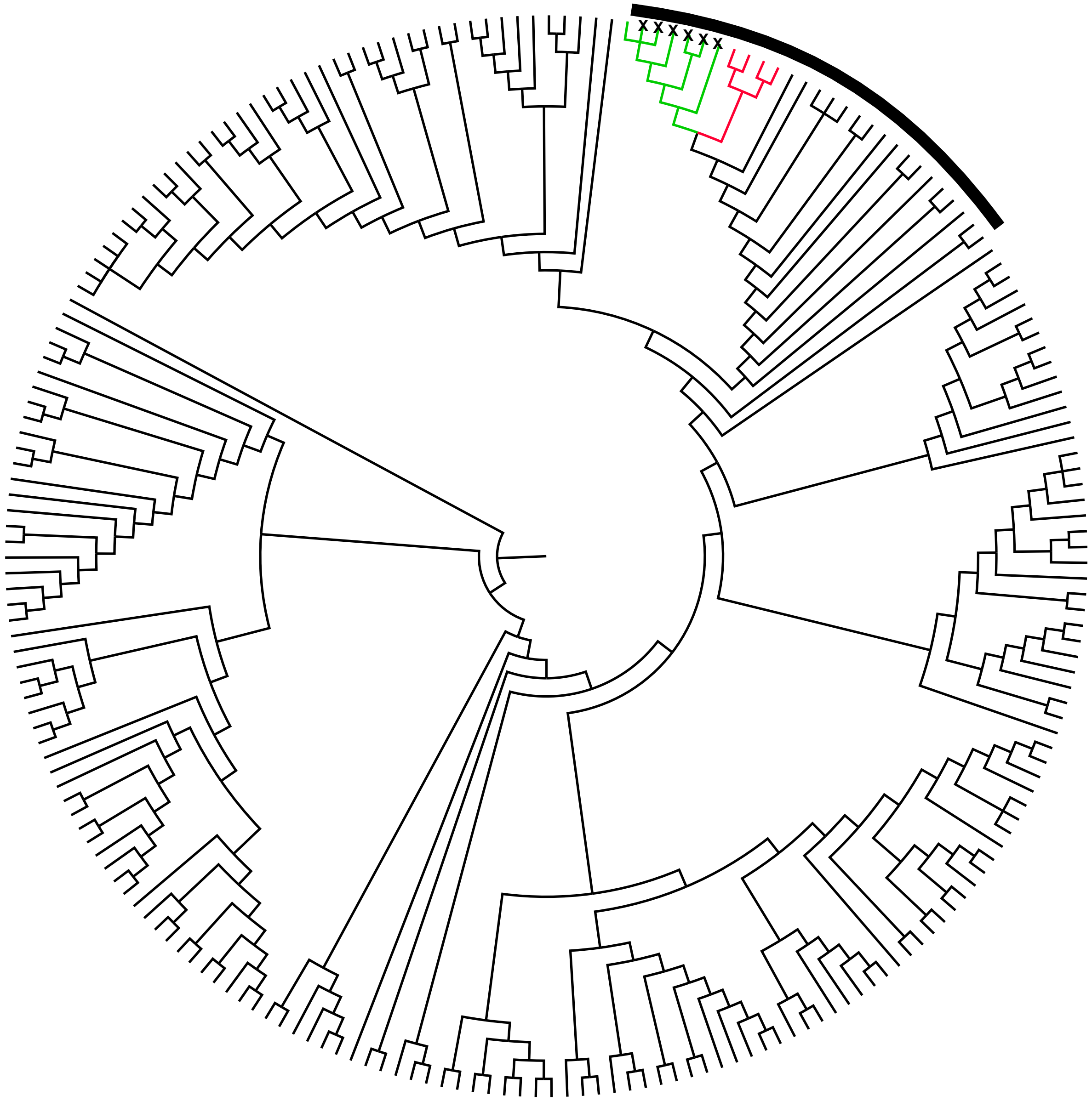

Figure S7. Nuclear phylogenies of *Ipomoea* Clade A3 indicating the position of the different tetraploid entities. (a) Maximum Likelihood (IQ-Tree), numbers on the branches indicate ultrafast bootstrap support (1,000 replicates). (b) Approximate Maximum Likelihood (FastTree2), numbers on the branches indicate Shimodaira-Hasegawa-like support values. (c) Species coalescent (Astral III), numbers on the branches indicate percentage of quartets that support the main topology and the two alternative topologies. Green, hexaploid *I. batatas*; red, *I. trifida*; blue, *I. aequatoriensis*; yellow, modern hybrids *I. tabascana* and *I. batatas* var. *apiculata*. Black dots indicate 100% support.

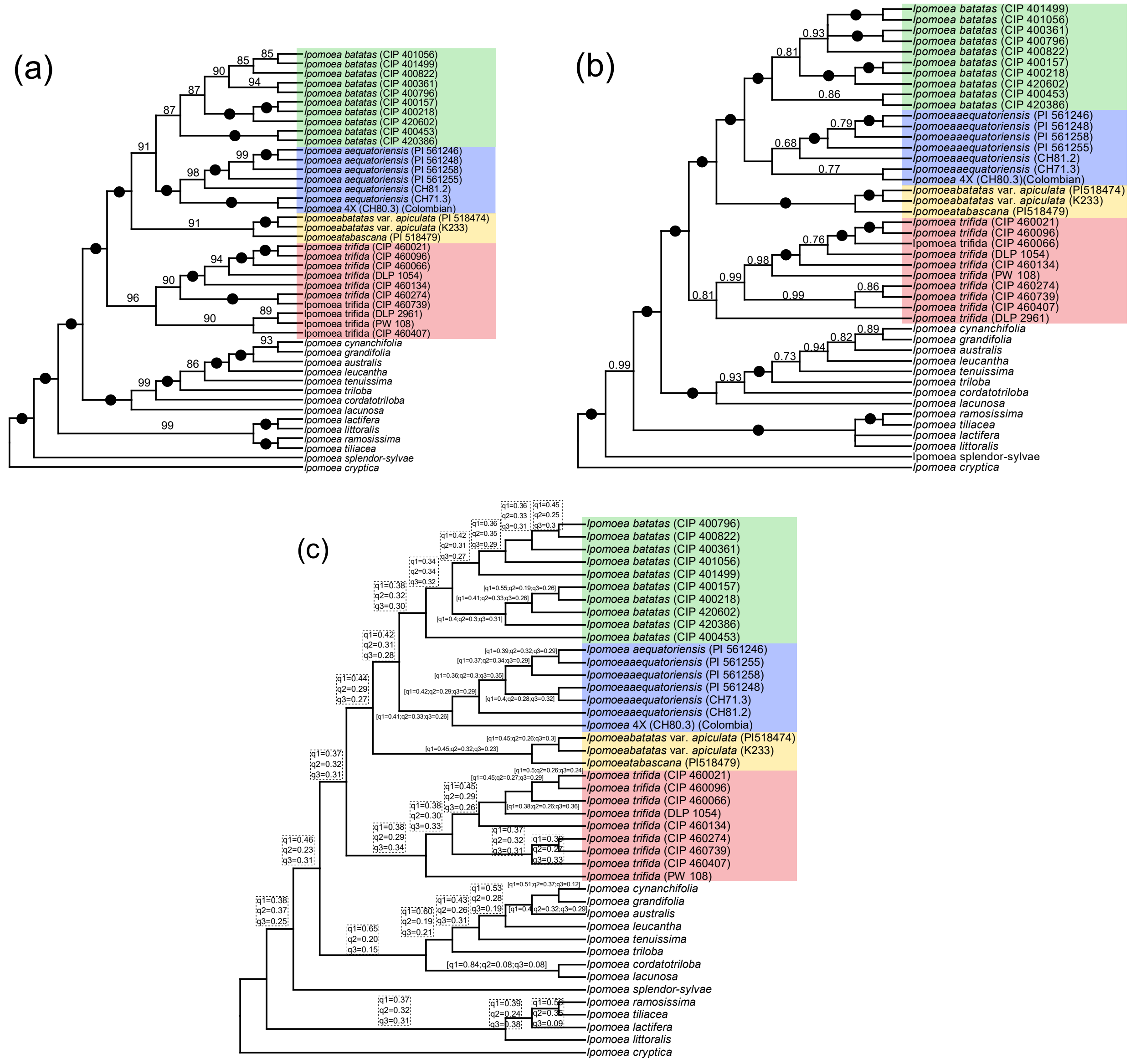

Figure S8. Nuclear phylogenies of *Ipomoea* Clade A3 indicating the position of the different tetraploid entities. Phylogenies inferred from consensus sequences including IUPAC ambiguous characters for heterozygous sites. (a) Species coalescent (Astral III), numbers on the branches indicate percentage of quartets that support the main topology and the two alternative topologies. (b) Approximate Maximum Likelihood (FastTree2), numbers on the branches indicate Shimodaira-Hasegawa-like support values. (c) Maximum Likelihood (IQ-Tree), numbers on the branches indicate ultrafast bootstrap support (1,000 replicates). Green, hexaploid *I. batatas*; red, *I. trifida*; blue, *I. aequatoriensis*; yellow, modern hybrids *I. tabascana* and *I. batatas* var. *apiculata*. Black dots indicate 100% support.

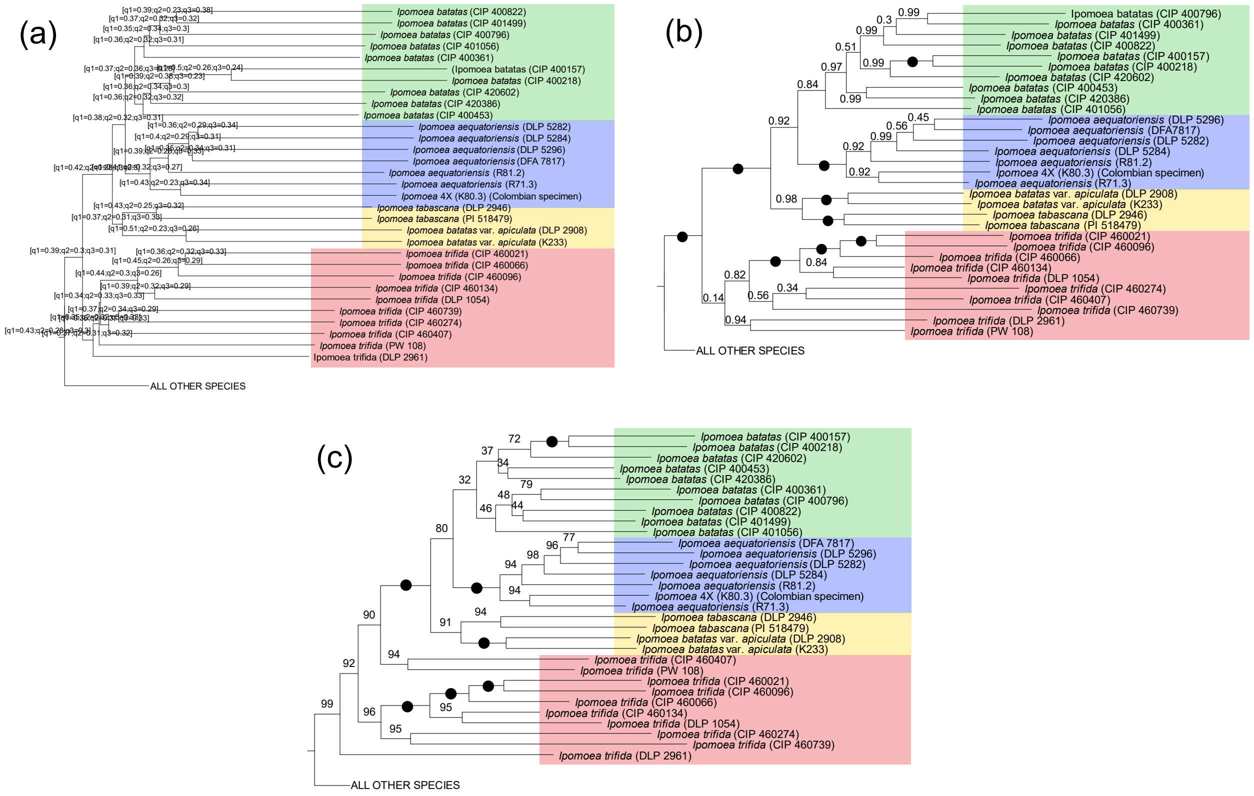

Figure S9. Additional Principal Component Analyses inferred using nuclear SNPs and all *Ipomoea trifida* (red dots) and *I. batatas* (green) samples in addition to *I. aequatoriensis* (blue) and the hybrids *I. tabascana* (black) and *I. batatas* var. *apiculata* (orange). (a) PCA inferred without a previous LD-pruning step using 914 SNPs. (b) PCA inferred after an LD-pruning step using 764 SNPs. Ellipses indicate normal distribution of the data. Light blue point indicates the Colombian specimen K500/CH80.3 discussed throughout the text.

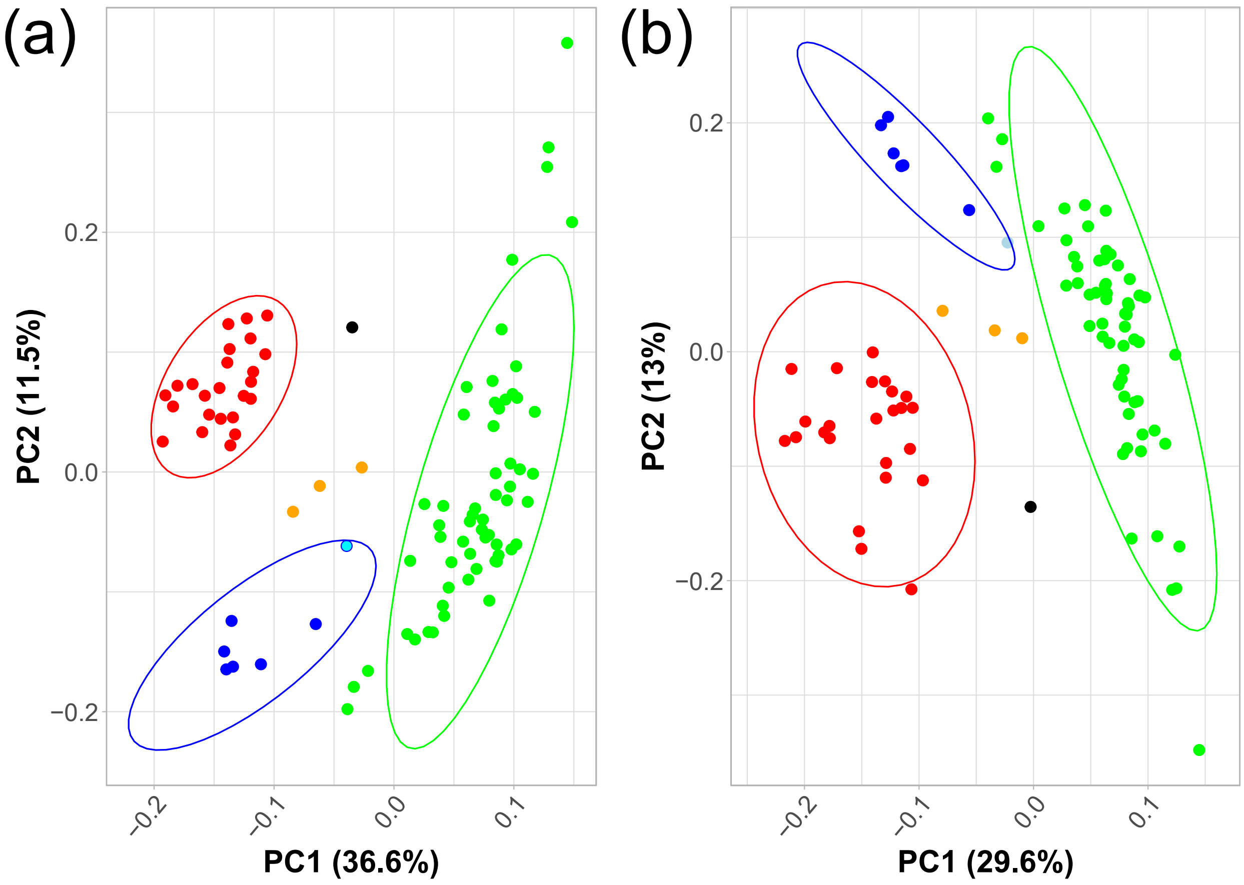

Figure S10. Illustration of *Ipomoea aequatoriensis*, showing: (a) habit with entire leaves and inflorescence; (b) stem with trilobed leaves; (c) leaf base, adaxial surface; (d) leaf base, abaxial surface with pair of glands; (e) sepals, outer (left) to inner (right); (f) corolla opened up to show stamens; (g) ovary and style. Drawn by Rosemary Wise from *Sparre* 15308 (A, C, D and E) and *Austin* 7817 (B, F and G).

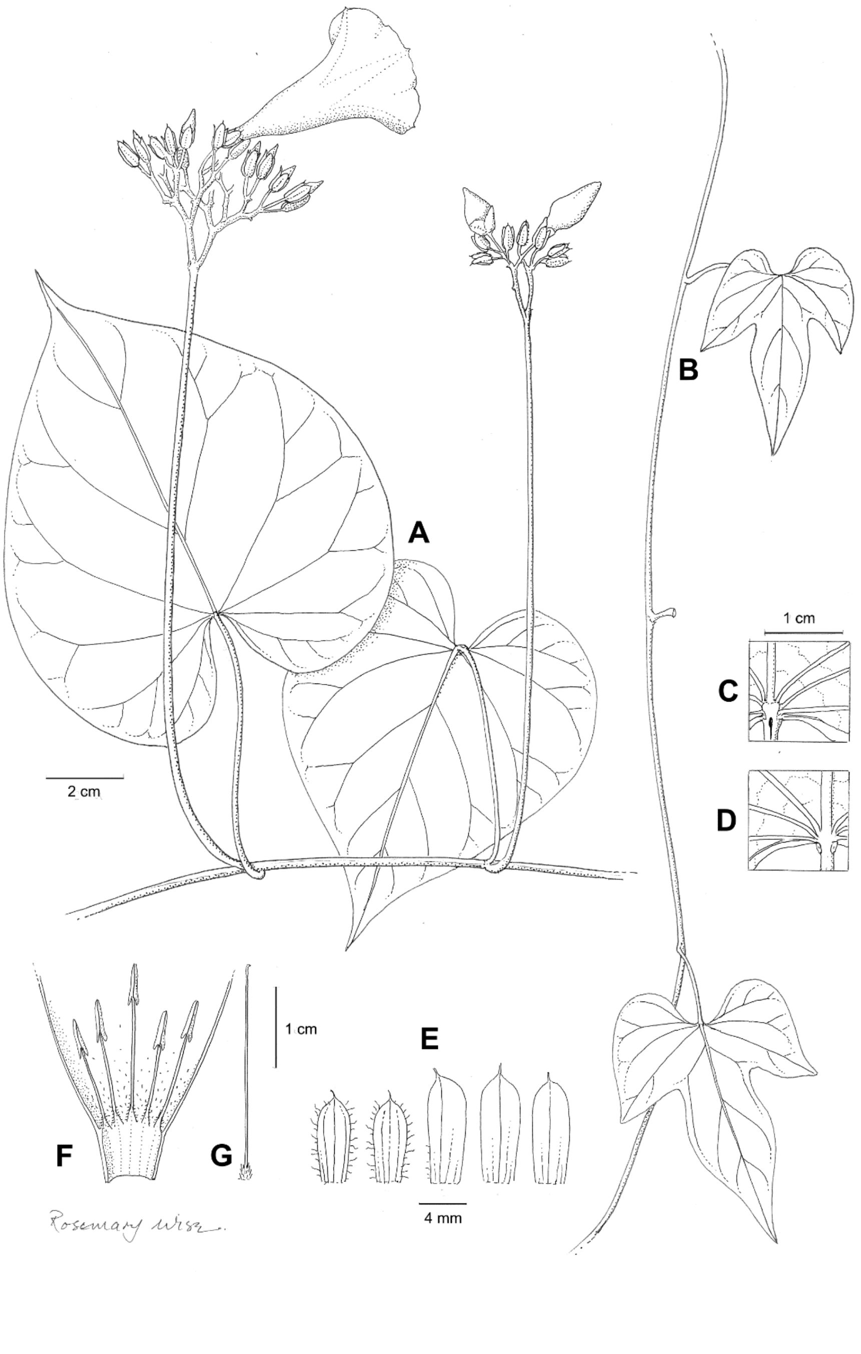

Table S5. Patterns of nucleotide heterozygosity in k-mer spectra of sequencing reads (k=21). All samples tetraploid.

Species	Sample	Origin	Haploid length (bp)	Heterozygosity (%)	aaab (%)	aabb (%)	Read errors (%)
I. aequatoriensis	PI561248	Ecuador	609,771,573	3.67	2.28	1.04	0.0746
I. batatas var. *apiculata*	K233	Mexico	456,325,693	12	0.001	10.2	0.344
I. batatas var. *apiculata*	PI518474	Mexico	508,703,597	11.4	0.001	9.37	0.207
I. tabascana	PI518479	Mexico	502,475,789	11.9	0.001	9.62	0.322

METHODS S1. Preliminary analysis of the *trnL-rpl32 chloroplast DNA region*

The preliminary phylogeny using the *trnL-rpl32* chloroplast DNA region included 215 species representing all main lineages in *Ipomoea* (Muñoz-Rodríguez *et al.*, 2019) and one *Operculina pteripes* (G.Don) O'Donell sample as outgroup (Table S2). We obtained new DNA extractions from 7 Ecuadorian and 1 Colombian specimens, whereas sequences for the other species were generated in a previous study and are available via GenBank (see Table S2).

We extracted DNA from herbarium samples using the Plant Tissue Mini protocol for QIAGEN DNEasy Plant Mini Kit. We used custom primers for amplification (VR27-F: 5'-GTAATACAATAAGGCGGATA; VR27-R: 5'-ATTACATGACAAGATAGTCTTG) with a reagent volume of 15 µl (7.3 µl H_2O, 3 µl buffer, 0.7 µl $MgCl_2$, 0.3 µl of each primer diluted to a concentration of 10X, 0.5 µl dNTPs, 1 µl BSA, 0.4 µl Taq polymerase, 1.5 µl sample DNA) and standard PCR conditions (5' at 80°C; 30 cycles of 1' at 95°C, 1' at 50°C, and 4' at 65°C and a final stage of 4' at 65°C). We cleaned the PCR reactions using the GeneJET PCR purification kit and sequenced the samples using Sanger sequencing at Source Bioscience.

We aligned the sequences using MAFFT v.7.310 (Katoh & Standley, 2013) and edited the alignment in Geneious to remove all position with 25% gaps or more. We then inferred an Approximate Maximum Likelihood phylogeny using FastTree 2.1.10, GTR+Gamma model (Price *et al.*, 2010).

METHODS S2. Selection of *Ipomoea* probes for nuclear phylogenetic analysis

Our analysis of nuclear DNA data utilized a subset of the nuclear probes used in a study on the origin of sweet potato published in 2018 (Muñoz-Rodríguez *et al.*, 2018). In this section we

present a detailed account of how the probes were generated. In 2014, we developed probes targeting 605 putative single copy nuclear regions of *Ipomoea* through comparison of genomic data from *I. lacunosa* and CDS regions of *Solanum tuberosum*. Regions between *Ipomoea* and *Solanum* with a one-to-one match at 70% identity along at least half the length of a Solanum CDS were filtered to retain Ipomoea loci that were at least 1000 bp. Along these loci, a set of 20,020 100 bp custom RNA baits were developed by MycroArray (Ann Arbor, MI), excluding probes with GC content < 25%. These 20,020 baits matched unique positions in the scaffolded *I. lacunosa* genome and resulted in 605 preliminary *Ipomoea* probes, 1,018–9,646 bp long, located in 506 different scaffolds.

Recently published high-quality genomes of diploid relatives of sweet potato (Hoshino *et al.*, 2016; Wu *et al.*, 2018; Li *et al.*, 2019) allowed a broader comparison with two other species in the sweet potato group, *I. trifida* and *I. triloba*. An initial Blast search revealed that 217 of our 605 original probes overlapped at least partially with others, hence indicating that the set of probes in our previous study included a number of potentially duplicate regions that could have an effect in the phylogenetic trees inferred. We believe these to have originated from separate scaffolds of the partially assembled *I. lacunosa* genome that we used to design the bait set in 2014. We therefore cleaned the dataset and retained only the 388 probes that we were confident are truly single copy. We further mapped these 388 probes to all *Ipomoea* genomes available as of October 2021 using Bowtie2 (end-to-end, -k 2, L = 15) (Langmead & Salzberg, 2012) and found that two of them align to more than one place in at least two of those genomes, hence removed them from the final set of probes. In summary, we used a set of 386 *Ipomoea* nuclear DNA probes that we are confident are single-copy regions in several species closely related to sweet potato. We used these regions for phylogenetic analysis of nuclear DNA data in this study.

METHODS S3. Additional phylogenetic analyses of nuclear probes

The analysis of nuclear probes presented in the main Methods section of this paper used only homozygous sites from the assemblies and discarded heterozygous sites. In addition, we also inferred additional analyses coding the heterozygous sites using ambiguous IUPAC characters. While programmes such as FastTree treat ambiguous IUPAC characters as Ns, IQ-Tree supports them, giving each represented character equal likelihood (http://www.iqtree.org/doc/Frequently-Asked-Questions). Other possibilities for generating the consensus offered by BCFtools *consensus*, such as always assigning the alternative allele in the consensus sequence (the default option) seemed less appropriate in our case.

We thus repeated the analysis of consensus sequences, this time including IUPAC

ambiguous characters, using partitioned ML analysis of concatenated alignments with automated model selection + merge in IQ-Tree 1.6.12 (Nguyen *et al.*, 2015; Kalyaanamoorthy *et al.*, 2017), and independent gene tree inference using IQ-Tree 1.6.12 with automated model selection followed by species tree inference using the coalescent in Astral III (Zhang *et al.*, 2018). We also ran Approximate ML analysis of unpartitioned concatenated alignments in multi-threaded double-precision FastTree 2.1.10[54,71] (GTR + gamma model), but since FastTree treats IUPAC ambiguous characters as Ns, the results (not shown) were the same as in the analysis using only homozygous sites.

Phylogenies inferred using this approach have the same topology as phylogenies inferred using homozygous sites only (Figure S8).

METHODS S4. Additional Principal Component analyses

The PCA analysis presented in the main Methods section used a subset of samples of *I. batatas* and *I. trifida* to avoid bias due to different population sizes (Privé *et al.*, 2020). Nevertheless, we also ran two additional analyses using all *I. batatas* and *I. trifida* samples and including or not a linkage disequilibrium pre-pruning step. These analyses used 912 (no LD pruning) and 774 SNPs (with LD pruning) and the results are consistent with the analysis using a reduced sampling (Figure S9b).

METHODS S5. K-mer analysis of putative hybrid tetraploids

We also tested GenomeScope2.0 with three samples of putative hybrid origin, namely one *Ipomoea tabascana* (PI 518479) and two *I. batatas* var. *apiculata* (PI 518474 and K233). These samples had lower coverage (~30X) than the one Ecuadorian sample analysed.

NOTES S1. Modern hybrids closely related to *Ipomoea batatas*

Two tetraploid entities associated with *I. batatas* have been described: *Ipomoea tabascana* J.A.McDonald & D.F.Austin and *I. batatas* var. *apiculata* J.A.McDonald & D.F.Austin. Both entities were described in 1990, following fieldwork in Mexico by Daniel Austin a few years earlier (McDonald & Austin, 1990). According to the original publication where both entities were described, Daniel Austin collected one specimen of each in 1987. In a later visit (1991) to the site in search of *I. tabascana*, the authors found no seeds and reported no new collections (Austin *et al.*, 1991), and in 1995 they collected one seed (Contreras *et al.*, 1995), which possibly is the original sample from which all subsequent studies including this species obtained their material. The species has not been collected since then. In other words, *I. tabascana* is formally only known from a single collection and one seed, and no living populations have been reported in three decades. In subsequent studies, it was shown to be most likely of hybrid origin (Srisuwan, 2006; Muñoz-Rodríguez *et al.*, 2018).

Similarly, *Ipomoea batatas* var. *apiculata* has only been collected 5 times since 1845, all of them from the same locality in the vicinity of Veracruz, and Austin and collaborators treated it as "feral", rather than wild. Its likely hybrid origin is shown in this paper. Our results support the recognition of this variety as another distinct entity of hybrid origin instead of a variety of *Ipomoea batatas*, and thus the name *Ipomoea apiculata* M.Martens & Galeotti may be more appropriate. We refrain from formally proposing this change here as ongoing studies will confirm whether this entity is distinct from or conspecific with *Ipomoea tabascana* and the other tetraploid plants.

In summary, the very few specimens available for these two entities and the fact that they all likely come from the same locality raises doubts about their status. It is also not clear whether they represent stable tetraploid progeny or one-off occurrences. Further field explorations may clarify this question.

NOTES S2. Results k-mer analyses using GenomeScope2.0

PI 561248 ECUADOR

p = 4
k = 21
max_kmercov = 500

property	min	max
Homozygous (aaaa)	93.4414%	97.7768%
Heterozygous (not aaaa)	2.2232%	6.55857%
aaab	2.03645%	2.52079%
aabb	0.186755%	1.88815%
aabc	0%	1.49173%
abcd	0%	0.657907%
Genome Haploid Length	596,335,479 bp	609,771,573 bp
Genome Repeat Length	153,231,028 bp	156,683,491 bp
Genome Unique Length	443,104,452 bp	453,088,082 bp
Model Fit	85.268%	97.8294%
Read Error Rate	0.0746074%	0.0746074%

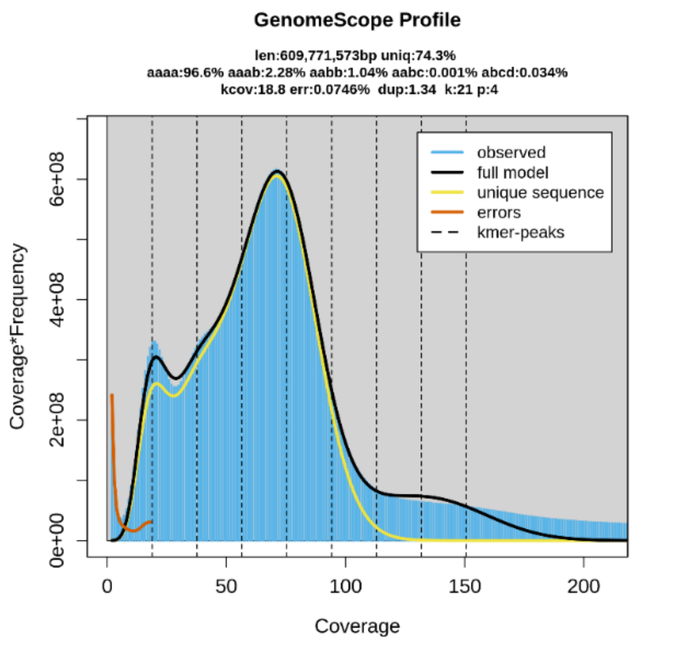

K233 MEXICO

p = 4k = 21
max_kmercov = 100000

property	min	max
Homozygous (aaaa)	72.033%	92.6982%
Heterozygous (not aaaa)	7.30181%	27.967%
aaab	0%	4.77248%
aabb	7.11871%	13.2002%
aabc	0%	6.59087%
abcd	0.183092%	3.40347%
Genome Haploid Length	450,538,829 bp	456,325,693 bp
Genome Repeat Length	242,752,389 bp	245,870,377 bp
Genome Unique Length	207,786,441 bp	210,455,316 bp
Model Fit	54.027%	92.1498%
Read Error Rate	0.343842%	0.343842%

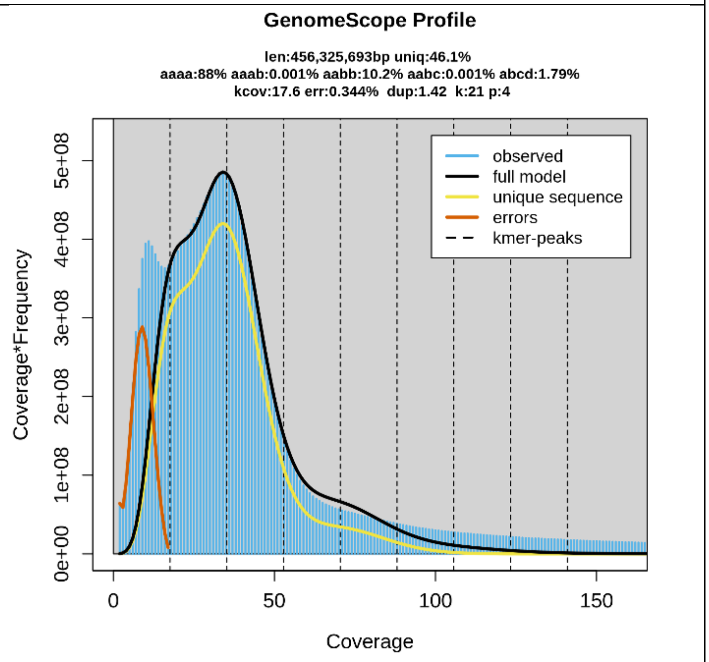

PI 518474 MEXICO

p = 4k = 21
max_kmercov = 100000

property	min	max
Homozygous (aaaa)	68.6825%	95.2674%
Heterozygous (not aaaa)	4.73264%	31.3175%
aaab	0%	2.11278%
aabb	4.73264%	13.9981%
aabc	0%	10.0273%
abcd	0%	5.17934%
Genome Haploid Length	501,331,466 bp	508,703,597 bp
Genome Repeat Length	271,828,231 bp	275,825,494 bp
Genome Unique Length	229,503,235 bp	232,878,104 bp
Model Fit	51.9357%	92.8567%
Read Error Rate	0.207173%	0.207173%

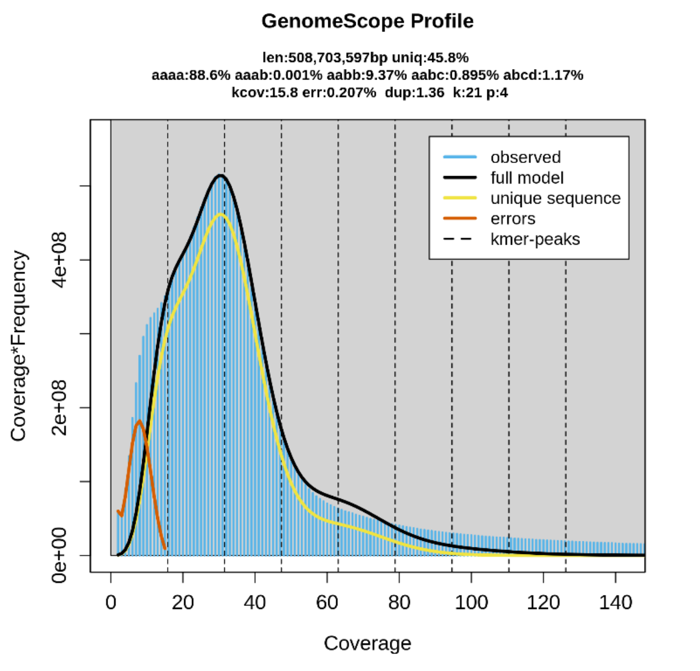

PI 518479 MEXICO

p = 4k = 21
max_kmercov = 100000

property	min	max
Homozygous (aaaa)	70.4436%	94.3532%
Heterozygous (not aaaa)	5.64682%	29.5564%
aaab	0%	4.35383%
aabb	5.64682%	13.5952%
aabc	0%	8.04151%
abcd	0%	3.56586%
Genome Haploid Length	495,610,302 bp	502,475,789 bp
Genome Repeat Length	281,190,703 bp	285,085,922 bp
Genome Unique Length	214,419,599 bp	217,389,866 bp
Model Fit	49.5486%	92.6182%
Read Error Rate	0.321655%	0.321655%

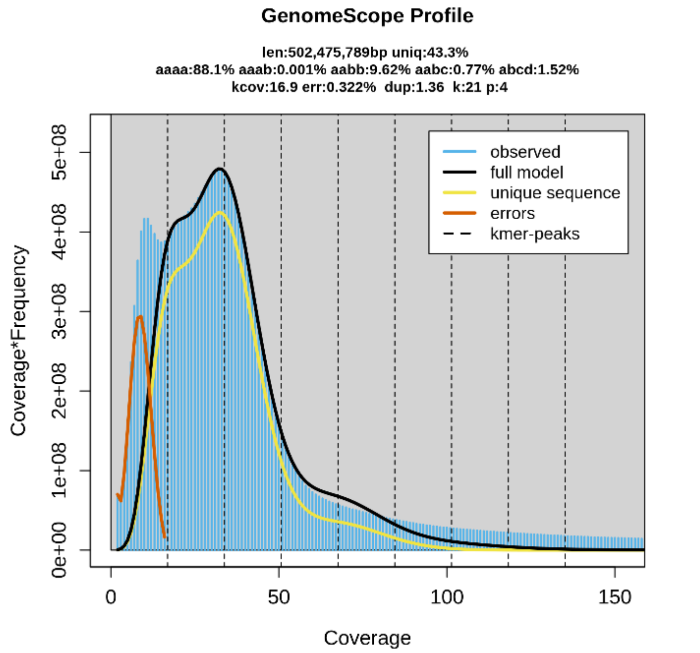

NOTES S3. Description and additional information on *Ipomoea aequatoriensis*

Description:

Perennial twining herb to 3 metres. Roots fibrous or somewhat thickened to 1cm diam. (Figures S1-S4); storage roots not seen. Stems 1–3 (–4) mm diam., grey-green, drying reddish-brown on exposed surface, glabrous or thinly to densely pilose, especially at nodes, hairs sometimes asperous. Leaves petiolate, 4–14 × 3–13.5 cm, ovate, entire or, less commonly, shallowly 3-lobed or intermediate between the two states (Figures 4A-B); apex shortly acuminate to cuspidate, with obtuse, mucronate tip; base broadly cordate; margins subentire to undulate, weakly-developed lateral lobes sometimes present; veins palmately divided with prominent central rib; green adaxially, paler abaxially; glabrous to thinly pubescent on both surfaces, often with hairs along veins (Figures 4C-D); petioles 2–12.5 cm, thinly to densely pilose, with a pair of glands at junction with leaf base (Figure 4D). Inflorescence axillary, formed of solitary, pedunculate, usually dense, umbellate cymes with 5-24 flowers; peduncles straight to slightly curved, rarely forked upwards, (3–)9–30 cm, glabrous or thinly pilose; short secondary and tertiary peduncles generally present, 4-10 mm, noticeably thicker than pedicels; bracteoles c. 1–1.5 x 0.5 mm, ovate-deltoid, acuminate, chartaceous, caducous; pedicels (3–)6–12(–17)mm, slender, glabrous; sepals unequal, oblong-elliptic to obovate, obtuse and mucronate (Figure 4E), the central vein prominent, often with a distinct fold below apical part, outer pair 3–7 x 2–2.5 mm long, ciliate, inner three 7–10 x 3–3.5 mm, somewhat chartaceous on margins, glabrous; corolla 3–4.5 cm long, funnel-shaped, pale pink with darker centre, glabrous on the exterior; stamens unequal, two long and three shorter (Figure 4F), glabrous except glandular-hairy base; longest filament c. 15 mm, shortest filament c. 6 mm; anthers 2.5 mm long, linear with sagittate base; style c. 20 mm long, glabrous; ovary pubescent (Figure 4G). Capsules (observed only on *Asplund* 16545) c. 5 x 6 mm, appressed ovoid, pilose; seeds 3 x 2 mm, dark brown, glabrous.

Habitat:

Secondary forest, clearings, and riverside scrub. 0 – 750 m.

Distribution:

Coastal departments of Ecuador (El Oro, Esmeraldas, Guayas, Loja, Los Ríos, Manabí).

Etymology:

The name *aequatoriensis* is chosen for the distribution of the species, which grows on either side

of the Equator in Ecuador and possibly Colombia (see Notes below).

Additional specimens:

ECUADOR. El Oro: Arenillas, *Asplund, E.* 15676 (K, S); Atahualpa, *Cornejo, X.* 304 (GUAY, QCNE); El Guabo, *Austin, D. F. & De La Puente, F.* 7817/5298 (FTG; OXF); Machala, *Austin, D. F. & De La Puente, F.* 7820/5301 (FTG). **Esmeraldas:** Atacames**,** *Sparre, B.* 15341(S); Esmeraldas, *Asplund, E.* 16545 (S); *Austin, D. F. & De La Puente, F.* 7801/5282 (FTG); *Austin, D. F. & De La Puente, F.* 7802/5283 (FTG, CIP); *Balslev, H. et al.* 3131 (GB, QCA); *Hoover, W. S. et al.* 4129 (QCA); *Hudson, J. 730* (US, MO); Borbón, *Austin, D. F. & De La Puente, F.* 7803/5284 (FTG, CIP), *Austin, D. F. & De La Puente, F.* 7804/5285 (FTG, CIP); *Besse, L. et al.* 2305 (QCA); *Cornejo, X. & Bonifaz, C.* 1867 (GUAY); *Játiva, C. & Epling, E.* 1191 (S); Quininde, *Bass, M. S.* 208 (QCNE); *Rick, C. M.* SAM2614 (OXF); Rio Cayapa, *Holm-Nielsen, L. et al.* 25318 (ARIZ, AAU); *Kvist, L. P.* 40476 (QCA, QCNE); Tabuche, *Sparre, B.* 15517 (S); *Sparre, B. 15286* (S); *Sparre, B. 15308* (S). **Guayas:** Ayangue, *Madsen J.E.* 50113 (QCA, QCNE); Guayaquil, *Austin, D. F. & De La Puente, F.* 7812/5293 (FTG); *Austin, D. F. & De La Puente, F.* 7815/5296 (FTG); Isla Puná, *Madsen, J. E.* 64032 (GUAY, QCA, QCNE); Montanita, *Cornejo, X. & Bonifaz, C.* 2283 (AAU, GUAY); Naranjal, *Austin, D. F. & De La Puente, F.* 7816/5296 (FTG); San Ignacio, *Holmgren, I* 88 (S). **Loja:** Cangonoma, *Austin, D. F. & De La Puente, F.* 7821/5302 (FTG); Puyango, *Austin, D. F. & De La Puente, F.* 7823/5304 (FTG); *Rick, C. M.* SAM5316 (OXF). **Los Ríos:** Rio Palenque, *Dodson, C. H. & McMahon, M. P.* 5112 (QCA); *McMahon, M. P.* 4207 (QCA); *Dodson, C. H. & Vrieze, J. M.* 4334 (QCA). **Manabí:** Jipijapa, *Montesdeoca, M. et al.* 677 (QAP); *Cerón, C. E. et al.* 18775 (OXF, QAP); San Placido, *Harling, G. & Andersson, L.* 24997 (QCA).

Notes:

The isotype specimen at CIP is also annotated with a separate collection number for Fermín de la Puente (5284). Several plants from Colombia resemble the Ecuadorian material, and the only specimen sequenced in this study is recovered with it in a strongly supported clade in phylogenetic analysis of nuclear sequence data (Figs. 2a, S7 and S8). Chloroplast data suggests, however, that a degree of introgression from hexaploid *I. batatas* may have occurred (Fig. 3). These Colombian specimens are not coastal but found at higher altitudes (up to 2000m), and there is an apparent gap in the distribution in southern Colombia (Figure 2b), although this could simply be the result of less collecting in the area.

COLOMBIA. Caldas: Risaralda, *Alvarez et al.* 5 (E, OXF); **Valle del Cauca:** Cali, *Kobayashi, M.* K500 (OXF); Calima, *Hugh-Jones, D. L.* 25 (K).

NOTES S4. HYBRID SPECIMENS IN OTHER STUDIES

We have not been able to analyse all the material listed in earlier studies of tetraploid plants (Table S1). However, comparison of the phylogenetic results presented here with those presented by Yan *et al.* (2021) indicates that the material they designate as the “basal *I. batatas* 4x lineage” (accessions CIP 403270, CIP 695141, CIP 695150 and PI 518474) is most likely of modern hybrid origin, rather than being the tetraploid progenitor of hexaploid *I. batatas* as they suggest. This is supported by the fact that accession PI 518474 is a specimen collected by Fermín de la Puente with number 2908. Identified as *I. batatas* var. *apiculata,* that specimen is also included in our study and shown to be a likely modern hybrid. Meanwhile, no collection details or material were available on request for CIP 695141 or CIP 695150, but both accessions have previously been identified as *I. trifida* (Srisuwan *et al.*, 2006), and appear to form part of a crop improvement breeding programme at CIP (pers. comm., CIP germplasm bank). Collection details are not available for these specimens, but there are no reports of tetraploid material having been collected in Peru, and the distribution of *I. trifida* is restricted to Central America, the Caribbean and the northern coasts of Colombia and Venezuela. These specimens are therefore most likely to have originated through controlled crosses at CIP. Our results thus highlight the importance of comprehensive studies in differentiating between the various tetraploid entities connected with hexaploid sweetpotato and of accurately inferring their role in its origin.

SUPPLEMENTARY REFERENCES

Austin DF, de la Puente F, Contreras J. **1991**. *Ipomoea tabascana*, an endangered tropical species. *Economic Botany* **45**: 435.

Austin DF, Jarret RL, Tapia C, de la Puente F. **1993**. Collecting tetraploid *I. batatas* (L.) Lam. in Ecuador. *FAO/IBPGR Plant Genetic Resources Newsletter* **91/92**: 33–35.

Contreras J, Austin DF, De la Puente F, Díaz J. 1995. Biodiversity of sweet potato (*Ipomoea batatas*, Convolvulaceae) in Southern Mexico. *Economic Botany* **49**: 286-296.

Hoshino A, Jayakumar V, Nitasaka E, Toyoda A, Noguchi H, Itoh T, Shin-I T, Minakuchi Y, Koda Y, Nagano AJ, *et al.* 2016. Genome sequence and analysis of the Japanese morning glory *Ipomoea nil*. *Nature Communications* **7**: 13295.

Kalyaanamoorthy S, Minh BQ, Wong TKF, von Haeseler A, Jermiin LS. **2017**. ModelFinder: fast model selection for accurate phylogenetic estimates. *Nature Methods* **14**: 587–589.

Katoh K, Standley DM. **2013**. MAFFT multiple sequence alignment software version 7: improvements in performance and usability. *Molecular Biology and Evolution* **30**: 772–780.

Langmead B, Salzberg SL. **2012**. Fast gapped-read alignment with Bowtie 2. *Nature Methods* **9**: 357–359.

Li M, Yang S, Xu W, Pu Z, Feng J, Wang Z, Zhang C, Peng M, Du C, Lin F, *et al.* 2019. The wild sweetpotato (*Ipomoea trifida*) genome provides insights into storage root development. *BMC Plant Biology* **19**: 119.

McDonald JA, Austin DF. **1990**. Changes and additions in *Ipomoea* sect. *Batatas*. *Brittonia* **42**: 116–120

Muñoz-Rodríguez P, Carruthers T, Wood JRI, Williams BRM, Weitemier K, Kronmiller B, Ellis D, Anglin NL, Longway L, Harris SA, *et al.* 2018. Reconciling conflicting phylogenies in the origin of sweet potato and dispersal to Polynesia. *Current Biology* **28**: 1246-1256.e12.

Muñoz-Rodríguez P, Carruthers T, Wood JRI, Williams BRM, Weitemier K, Kronmiller B, Ellis D, Anglin NL, Longway L, Harris SA, *et al.* 2019. A taxonomic monograph of *Ipomoea* integrated across phylogenetic scales. *Nature Plants* **5**: 1136–1144.

Nguyen L-T, Schmidt HA, von Haeseler A, Minh BQ. **2015**. IQ-TREE: a fast and effective stochastic algorithm for estimating Maximum-Likelihood phylogenies. *Molecular Biology and Evolution* **32**: 268–274.

Price MN, Dehal PS, Arkin AP. **2010**. FastTree 2 – Approximately Maximum-Likelihood trees for large alignments (AFY Poon, Ed.). *PLoS ONE* **5**: e9490.

Privé F, Luu K, Blum MGB, McGrath JJ, Vilhjálmsson BJ. 2020. Efficient toolkit implementing best practices for principal component analysis of population genetic data. *Bioinformatics* **36**: 4449-4457.

Srisuwan S, Sihachakr D, Siljak-Yakovlev S. **2006**. The origin and evolution of sweet potato (*Ipomoea batatas* Lam.) and its wild relatives through the cytogenetic approaches. *Plant Science* **171**: 424–433.

Wu S, Lau KH, Cao Q, Hamilton JP, Sun H, Zhou C, Eserman L, Gemenet DC, Olukolu BA, Wang H, *et al.* 2018. Genome sequences of two diploid wild relatives of cultivated sweetpotato reveal targets for genetic improvement. *Nature Communications* **9**: 4580.

Yan M, Li M, Moeinzadeh M-H, Quispe-Huamanquispe DG, Fan W, Nie H, Wang Z, Heider B, Jarret R, Kreuze J, *et al.* 2021. Haplotype-based phylogenetic analysis uncovers the tetraploid progenitor of sweet potato. *ResearchSquare (preprint)*. https://doi.org/10.21203/rs.3.rs-750500/v1

Zhang C, Rabiee M, Sayyari E, Mirarab S. **2018**. ASTRAL-III: polynomial time species tree reconstruction from partially resolved gene trees. *BMC Bioinformatics* **19**: 153/
